# Forcing ATGL expression in hepatocarcinoma cells imposes glycolytic rewiring through PPAR-α/p300-mediated acetylation of p53

**DOI:** 10.1038/s41388-018-0545-0

**Published:** 2018-10-26

**Authors:** Luca Di Leo, Rolando Vegliante, Fabio Ciccarone, Illari Salvatori, Manuel Scimeca, Elena Bonanno, Andrea Sagnotta, Gian Luca Grazi, Katia Aquilano, Maria Rosa Ciriolo

**Affiliations:** 1 Department of Biology, University of Rome “Tor Vergata”, Via della Ricerca Scientifica 1, Rome, 00133 Italy; 2 Santa Lucia Foundation (IRCCS Fondazione Santa Lucia), Rome, 00143 Italy; 3 Department of Experimental Medicine and Surgery, University of Rome “Tor Vergata”, Via Montpellier 1, Rome, 00133 Italy; 4 IRCCS San Raffaele Pisana, Via della Pisana 235, Rome, 00163 Italy; 5 Digestive Surgery and Liver Unit, Santa Maria di Terni Hospital, Viale Tristano di Joannuccio, Terni, 05100 Italy; 6 Surgical and Medical Department of Translational Medicine, Sant’Andrea Hospital, Faculty of Medicine and Psychology, “Sapienza” University of Rome, Via di Grottarossa 1035-39, Rome, 00189 Italy; 7 Hepato-Pancreato-Biliary Surgery Unit, Department of Clinical and Experimental Oncology, Regina Elena National Cancer Institute—IRCCS, Via Elio Chianesi 53, Rome, 00144 Italy; 8 Present Address: The Microenvironmental Niche in Tumorigenesis and Targeted Therapy—MN3T, INSERM U1109HOPITAL CIVIL—Institut d’Hématologie et d’Immunologie 1, Place de l’Hôpital, 67091 Strasbourg Cedex, France

**Keywords:** Biochemistry, Cancer, Liver cancer

## Abstract

Metabolic reprogramming is a typical feature of cancer cells aimed at sustaining high-energetic demand and proliferation rate. Here, we report clear-cut evidence for decreased expression of the adipose triglyceride lipase (ATGL), the first and rate-limiting enzyme of triglyceride hydrolysis, in both human and mouse-induced hepatocellular carcinoma (HCC). We identified metabolic rewiring as major outcome of ATGL overexpression in HCC-derived cell lines. Indeed, ATGL slackened both glucose uptake/utilization and cell proliferation in parallel with increased oxidative metabolism of fatty acids and enhanced mitochondria capacity. We ascribed these ATGL—downstream events to the activity of the tumor-suppressor p53, whose protein levels—but not transcript—were upregulated upon ATGL overexpression. The role of p53 was further assessed by abrogation of the ATGL-mediated effects upon p53 silencing or in p53-null hepatocarcinoma Hep3B cells. Furthermore, we provided insights on the molecular mechanisms governed by ATGL in HCC cells, identifying a new PPAR-α/p300 axis responsible for p53 acetylation/accumulation. Finally, we highlighted that ATGL levels confer different susceptibility of HCC cells to common therapeutic drugs, with ATGL overexpressing cells being more resistant to glycolysis inhibitors (*e.g.*, 2-deoxyglucose and 3-bromopyruvate), compared to genotoxic compounds. Collectively, our data provide evidence for a previously uncovered tumor-suppressor function of ATGL in HCC, with the outlined molecular mechanisms shedding light on new potential targets for anticancer therapy.

## Introduction

Hepatocellular carcinoma (HCC) is the fifth most common and the second deadly human cancer worldwide [1]. Different risk factors have been associated with HCC occurrence, also related to variable geographic incidence, such as hepatitis B virus and hepatitis C virus infection, alcohol, aflatoxin B1, nonalcoholic steatohepatitis (NASH), and cirrhosis [1]. Radiofrequency, chemotherapy, resection and orthotropic liver transplantation are curative treatments advisable in the early stages of the disease [1], while patients in advanced stage(s) are recommended to be treated with Sorafenib, which exhibits a moderately significant increase in patient survival [2]. Therefore, HCC treatment is in need of more investigation and new therapeutic approaches.

Metabolic rewiring is a common feature of many cancer types and is considered as a strategy tumors use to sustain high-proliferation rate and energy demand, and an appealing target to counteract cancer progression and survival [3]. The most well-defined metabolic reprogramming of cancer cells is the Warburg effect, consisting in a switch from an oxidative to a glycolytic phenotype, also under normoxia, which enables most tumors to rapidly obtain glucose-derived energy and substrates for anabolic reactions [3]. The Warburg effect is widely observed in human cancers, including HCC, and often correlates with high-proliferation rate and aggressiveness [3, 4]. Besides glucose addiction, malignant cells largely rely on lipids, mostly fatty acids (FAs), both as energy source and as components of bio-membranes. Indeed, enhanced activity as well as expression of many enzymes involved in the de novo synthesis of FAs (*i.e.*, lipogenesis) has been associated with tumor occurrence and progression [5]. FAs in excess are stored as triglycerides (TGs) within specific cytoplasmic organelles, namely lipid droplets (LDs). Although adipose tissue is known as the most efficient lipid storage body district, LDs can be accumulated by all tissues, particularly in liver [6]. Upon proper stimuli, TGs depots are mobilized through a hydrolytic process referred to as lipolysis [7]. The *PNPLA2* gene-encoded adipose triglyceride lipase (ATGL) is the first and rate-limiting enzyme of the lipolytic cascade, being its catalytic activity harbored in the Ser47-Asp166 dyad [8, 9]. ATGL-mediated TGs hydrolysis culminates in the release of FAs and free glycerol due to the activity of the downstream lipases, namely hormone-sensitive lipase (HSL) and monoacylglycerol lipase (MAGL) [7]. Despite its first identification in adipose tissues [9], ATGL is also expressed in other districts, as demonstrated by massive lipid accumulation in ATGL-deficient mice tissues [10, 11]. Notably, ATGL activity impacts on several cell signaling pathways beyond metabolism. Indeed, ATGL-released FAs bind and activate nuclear receptors including the peroxisome proliferator-activated receptor-α (PPAR-α), thus impinging on a plethora of cellular processes ranging from inflammation to antioxidant defense [10, 12, 13]. During the last years, increasing attention has been paid to lipid catabolism alterations in cancer. Indeed, HSL and MAGL have already been showed to be implicated in many human cancers and associated comorbidities (*e.g.*, cachexia) [14, 15]. Remarkably, ATGL has been found significantly reduced in several human malignancies including lung and pancreatic cancer [16, 17]. Though it is well-known that hepatocyte homeostasis strongly relies on ATGL activity for TGs hydrolysis and FAs partitioning/signaling [18], no evidence supports ATGL as metabolic regulator in liver cancer. Moreover, ATGL has been demonstrated to have a protective role against steatohepatitis, a main risk factor for HCC occurrence [19–21]. On the basis of this, we hypothesized a role for ATGL in metabolic adaptation and signaling of HCC. Here, we provide evidence for ATGL downregulation in human HCC, which was also confirmed in an induced mouse model of HCC. Interestingly, we identified an ATGL/PPAR-α/p300 axis which resulted in acetylation and stabilization of the tumor-suppressor p53, which is also known to orchestrate the metabolic pathway [22]. By this mechanism, ATGL overexpression promoted a glycolytic-to-oxidative metabolic switch in HCC cells. In addition, we demonstrated that metabolic rewiring associated to ATGL conferred glycolysis inhibitor susceptibility to HCC cells. Overall, our data revealed a novel role for ATGL as tumor suppressor in HCC, which could be exploited for the development of personalized therapeutic strategies.

## Results

### ATGL is downregulated in human HCC and in a mice model of HCC

**Fig. 1:**
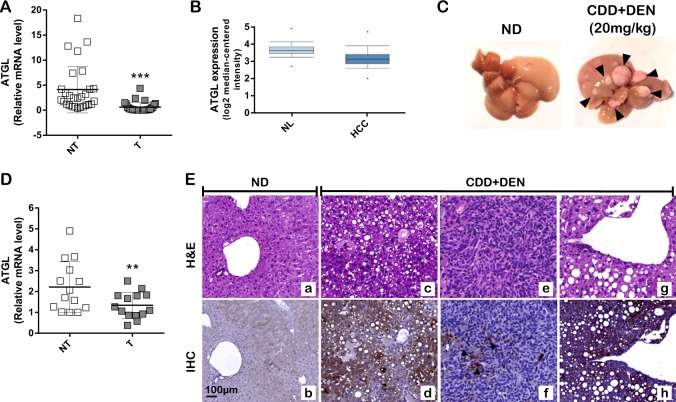
ATGL is downregulated in human HCC and in a mice model of HCC. **A** RT-qPCR analysis of *ATGL* expression in human HCC (T) *vs.* respective non-tumor counterparts (NT). *ACTB* was used as reference control (*n* = 28; ^***^p < *0.001* *vs.* NT). **B** *ATGL* expression was assessed by Gene Expression Omnibus through an Affymetrix Human Genome Array (>200 HCC *vs.* normal livers, NL), with a *p* value of 5.61E−21 and a fold change of −1.322. **C** Posteuthanasia representative images of livers from mice treated with ND and CDD + DEN. Tumor nodules are indicated with black arrows. **D** RT-qPCR analysis of *ATGL* expression in murine HCC nodules (T). Values were compared with the respective non-tumor counterparts (NT). *Actb* was used as reference control (*n* = 14; ^**^p < *0.01* *vs.* NT). **E** Morphology of livers and ATGL expression were respectively evaluated after H&E and IHC staining of histological sections obtained from ND and CDD + DEN treated mice. Representative images are reported. A well conserved liver architecture (panel a) and a faint signal for ATGL immunoreaction (panel b) was observed in ND treated mice. The parenchymal region distal from the cancer lesion of a mouse treated with CDD + DEN displayed fatty liver and a fully alteration of hepatic architecture (panel c) with numerous ATGL-positive hepatocytes (panel d). Image in panel e shows hepatic cancerous lesion with numerous spindle cells in a mouse treated with CDD + DEN. The cancerous mass displays rare ATGL-positive cancer cells (arrow heads) (panel f). Non-tumor parenchyma close to cancerous lesion was characterized by steatosis degeneration (panel g) and several ATGL-positive hepatocytes (panel h)

Driven by the evidence that ATGL is the major enzyme for hepatic TGs hydrolysis [18, 23] and that liver-specific ATGL deficiency causes hepatic steatosis [21], a contributing risk factor for HCC onset [24], the expression levels of the *PNPLA2* gene (hereinafter referred to as *ATGL*) were analyzed in human HCC biopsies. Twenty-eight HCC specimens were tested, and *ATGL* mRNA levels compared with the patient-matched non-tumor tissue. RT-qPCR results revealed a statistically significant reduction of the *ATGL* transcript in tumors (T) *vs.* non-tumor counterparts (NT) (Fig. 1A). This evidence was also confirmed by a publicly available dataset of human HCC samples (>200 HCC *vs.* normal livers) [25] (Fig. 1B). Results obtained in human specimens were corroborated in an induced mouse model of HCC (see Materials and methods). As soon as increased serum levels of the hepatic damage markers GOT-AST and GPT-ALT were observed in double-treated (CDD + DEN) *vs.* control (normal diet, ND) mice (at 45 weeks) (Supplementary Figure S1A, B), animals were sacrificed. A first morphological examination of livers displayed evident nodules compared to ND (Fig. 1C). A total of 14 nodules (T) with a diameter ≥3 mm were collected and their malignancy assessed by increased expression of the hepatocarcinoma marker α-fetoprotein (*Afp*) (Supplementary Figure S1C). Hence, *ATGL* expression was examined, resulting significantly decreased in T *vs.* NT (Fig. 1D). Histological analyses were performed as well, revealing altered hepatic parenchyma (mean score: 2.3 in CDD + DEN *vs*. 0.2 in ND; range: 0–3) and cancerous lesions in CDD + DEN mice (Fig. 1Ea, c, e, g). Importantly, cancerous lesions displayed negative-to-weak staining for ATGL (mean score of 0.5; range: 0–3) (Fig. 1Ef) *vs.* high-positive staining in the tumor-surrounding parenchyma (mean score = 2.3; range: 0–3) (Fig. 1Eh) and far-from-lesion parenchyma (mean score = 2.25; range: 0–3) (Fig. 1Ed). Weak—but still higher than tumors—positive-ATGL staining was observed in hepatocytes of ND mice (mean score = 1.4; range: 0–3) (Fig. 1Eb).

### ATGL levels negatively correlate with proliferation rate of HCC cell lines

**Fig. 2:**
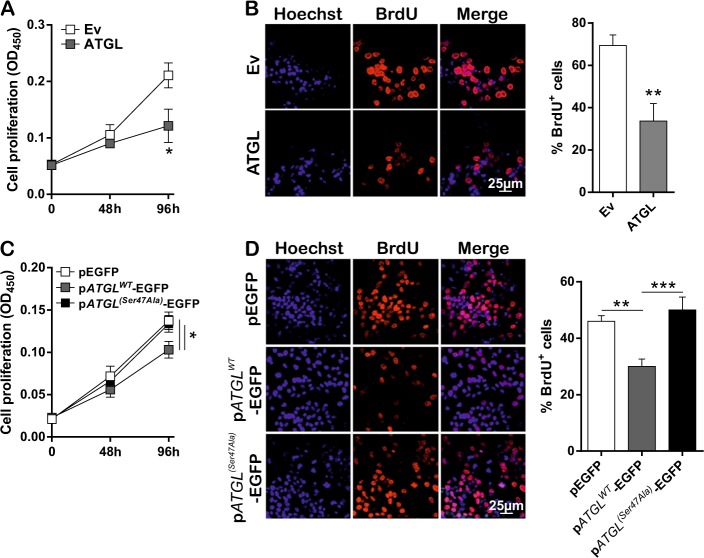
ATGL levels negatively correlate with proliferation rate of HCC cell lines. HepG2 cells were transfected for 48 and 96 h with pcDNA™4/HisMaxC-ATGL (ATGL) and pcDNA™4/HisMaxC (Ev), as negative control, plasmids. **A** Proliferation was assayed by CCK-8 colorimetric assay (*n* = 6; ^*^p < 0.05 *vs.* Ev) and **B** BrdU incorporation assay. Images reported in **B** are representative of *n* = 3 independent experiments. Bar graph refers to the percentage of BrdU-positive cells (*n* = 3; ^**^p < 0.01 *vs.* Ev). **C** HepG2 cells were transfected for 48 and 96 h with p*ATGL*^*WT*^-EGFP, p*ATGL*^*(Ser47Ala)*^-EGFP, and pEGFP plasmids and proliferation assayed by CCK-8 colorimetric assay (*n* = 6; ^*^p < *0.05* *vs.* pEGFP) and **D** BrdU incorporation assay. Images reported in **D** are representative of *n* = 3 independent experiments. Bar graph refers to the percentage of BrdU-positive cells (*n* = 3; ^**^p < 0.01; ^***^p < 0.001)

In order to assess whether ATGL downregulation has any effects on HCC growth, hepatoma HepG2 cells were transiently overexpressed with an ATGL-encoding plasmid (Supplementary Figure S2A) and their proliferation rate analyzed. A significant reduction of proliferation was observed upon ATGL overexpression, compared to the control (Ev, empty vector-transfected cells) as assessed by three different methods (Fig. 2A, B; Supplementary Figure S2B). Cell migration was also tested, but no change was disclosed (data not showed). Contrarily, a higher proliferation rate was noted when ATGL was silenced by siRNA, with respect to scrambled siRNA control cells (Supplementary Figure S2C).

We next investigated whether ATGL-mediated reduction of proliferation was linked to its catalytic activity. To this aim, HepG2 cells were transfected with plasmids coding for either wild-type ATGL (p*ATGL*^*WT*^-EGFP) or the ATGL mutant (p*ATGL*^*(Ser47Ala)*^-EGFP), bearing the substitution of an alanine for the catalytic serine 47 (Supplementary Figure S2D). Noticeably, no decrease in the proliferation rate was observed upon pATGL^*(Ser47Ala)*^-EGFP overexpression, as compared to the control (Fig. 2C, D; Supplementary Figure S2E).

ATGL-mediated reduced proliferation was confirmed in Huh7.5 (Supplementary Figure S2G–I), but not in Hep3B (Supplementary Figure S2J–L) cells upon ATGL overexpression (Supplementary Figure S2F).

### ATGL overexpression promotes glycolytic-to-oxidative metabolic switch in HCC cell lines

**Fig. 3:**
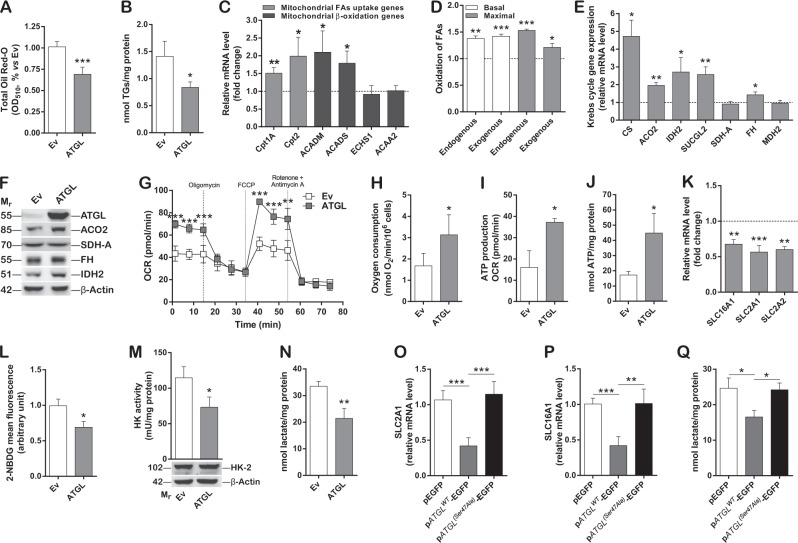
ATGL overexpression promotes glycolytic-to-oxidative metabolic switch in HCC cell lines. In all the experiments, HepG2 cells were transfected for 48 h. **A** ATGL activity was indirectly determined by measuring LDs content after Oil Red-O staining (*n* = 3; ^***^p < 0.001 *vs.* Ev). **B** TGs content was spectrophotometrically determined and relative absorbance normalized on total proteins (*n* = 3; ^*^p < 0.05 *vs.* Ev). **C** RT-qPCR analysis of mitochondrial FAs uptake (*Cpt1A* and *Cpt2*) and of mitochondrial β-oxidation (*ACADM*, *ACADS*, *ECHS1*, and *ACAA2*) genes. *ACTB* was used as reference control. Data are showed as fold change *vs.* Ev, represented by a dashed line (*n* = 6; ^*^p < 0.05; ^**^p < 0.01 *vs.* Ev). **D** Basal oxidation of endogenous FAs was calculated by subtracting basal oxygen consumption values, in the presence of XF BSA, to those under baseline conditions obtained in the presence of XF BSA-Etomoxir. Maximal oxidation of endogenous FAs was calculated as before but considering values obtained after FCCP injection. Similarly, basal and maximal oxidation of exogenous FAs was calculated taking as refence values obtained in presence of XF Palmitate-BSA and XF Palmitate-BSA-Etomoxir (*n* = 3; ^*^p < 0.05; ^**^p < 0.01; ***p < *0.001* *vs.* Ev, represented by a dashed line). **E** RT-qPCR analysis of Krebs cycle genes. *ACTB* was used as reference control. Data are graphed as in (**C**) (*n* = 6; ^*^p < 0.05; ^**^p < 0.01 *vs.* Ev). **F** Western blot analysis, representative of *n* = 3 independent experiments, of Krebs cycle enzymes. β-Actin and ATGL were used as loading and transfection controls, respectively. **G** OCR and **I** ATP production were determined by the Seahorse Bioscience XF^e^96 analyzer in combination with the Seahorse Bioscience XF Cell Mito Stress Test assay kit (*n* = 3; ^*^p < 0.05; ^**^p < 0.01; ^***^p < 0.001 *vs.* Ev). **H** Oxidative phosphorylation was monitored by measuring oxygen consumption by Clark’s electrode and **J** ATP content by luminometric assays (*n* = 4; ^*^p < 0.05 *vs.* Ev). **K** RT-qPCR analysis of *SLC16A1/MCT1*, *SLC2A1/GLUT1*, and *SLC2A2/GLUT2*. *ACTB* was used as reference control. Data are graphed as in (**C**) (*n* = 6; ^**^p < 0.01; ^***^p < 0.001 *vs.* Ev). **L** Cytofluorimetric analysis of glucose uptake by 2-NBDG. 10,000 events were counted (*n* = 3; ^*^p < 0.05 *vs.* Ev). **M** Spectrophotometric determination of hexokinase (HK) activity. Relative absorbance was normalized on total proteins (*n* = 3; ^*^p < 0.05 *vs.* Ev). Western blot refers to the levels of HK-2 isoform upon ATGL overexpression (*n* = 3). **N** Extracellular lactate content was measured after medium collection by an enzymatic/spectrophotometric combined technique (*n* = 6; ^**^p < 0.01 *vs.* Ev). **O** HepG2 cells were transfected for 48 h with a p*ATGL*^*WT*^-EGFP, a p*ATGL*^*(Ser47Ala)*^-EGFP and a pEGFP plasmid, as negative control, and *SLC2A1/GLUT1* and **P** *SLC16A1/MCT1* expression and **Q** extracellular lactate content evaluated as previously described (*n* = 3; ^*^p < 0.05; ^**^p < 0.01; ^***^p < 0.001)

Metabolism of cancer cells mainly relies on glycolysis to facilitate both proliferation and fast energy production [3]. Given the pivotal role of ATGL in TGs degradation and FAs release [20, 26], a possible contribution of ATGL downregulation to cancer metabolic reprogramming was investigated. Therefore, both oxidative and glycolytic pathways were analyzed in HepG2 cells. ATGL levels negatively correlated with LD and intracellular TGs content as demonstrated by either ATGL overexpression (Fig. 3A, B) or ATGL silencing (Supplementary Figure S3A). Moreover, we observed increased expression of several mitochondrial FAs uptake and β-oxidation genes (Fig. 3C), augmented long chain fatty acid oxidation (Fig. 3D) and no change in lipid biogenesis gene expression (Supplementary Figure S3B) upon ATGL overexpression, with respect to the control. In addition, we noted higher mRNA and protein levels of most Krebs cycle enzymes (Fig. 3E, F), in parallel with enhanced oxygen consumption (Fig. 3G, H; Supplementary Figure S3C–H) and ATP production and levels (Fig. 3I, J), speaking in favor of an overall augmented mitochondrial activity. Mitochondrial biogenesis and mass markers (Supplementary Figure S3I, J) as well as the expression of both nuclear and mitochondrial encoded electron transport chain genes (Supplementary Figure S3I–L) were not affected by ATGL overexpression, arguing for an optimized oxidative metabolism that was not related to mitochondrial content fluctuations. ATGL-driven LD degradation and enhanced mitochondrial activity were recapitulated in Huh7.5 (Supplementary Figure S3M–P) and Hep3B (Supplementary Figure S3Q–T) cells.

Next, we wondered whether ATGL-mediated enhancement of oxidative metabolism was a passive consequence of sustained TGs hydrolysis and FAs release fueling mitochondria or it was accompanied by changes in the glycolytic flux. Notably, ATGL levels negatively correlated with the expression of the lactate *SLC16A1/MCT1* and of the glucose *SLC2A1/GLUT1* and *SLC2A2/GLUT2* transporters in HepG2 cells, as demonstrated by either ATGL overexpression (Fig. 3K) or ATGL silencing (Supplementary Figure S4A). In addition, we noted diminished glucose uptake (Fig. 3L), hexokinase (HK) activity (Fig. 3M) and extracellular lactate concentration (Fig. 3N). The latter observation was significantly reverted upon ATGL silencing (Supplementary Figure S4B). ATGL effects on glucose metabolism were confirmed in Huh7.5 cells (Supplementary Figure S4C–E) but not in Hep3B cells (Supplementary Figure S4F–H).

Remarkably, p*ATGL*^*(Ser47Ala)*^-EGFP overexpression did not impact on either neutral lipid amount (Supplementary Figure S4I) or *SLC2A1/GLUT1* and *SLC16A1/MCT1* expression and extracellular lactate concentration (Fig. 3O–Q), suggesting that the catalytic activity of ATGL is required for the observed metabolic alterations.

### p53 is involved in ATGL-mediated metabolic adaptation and proliferation of HCC cell lines

**Fig. 4:**
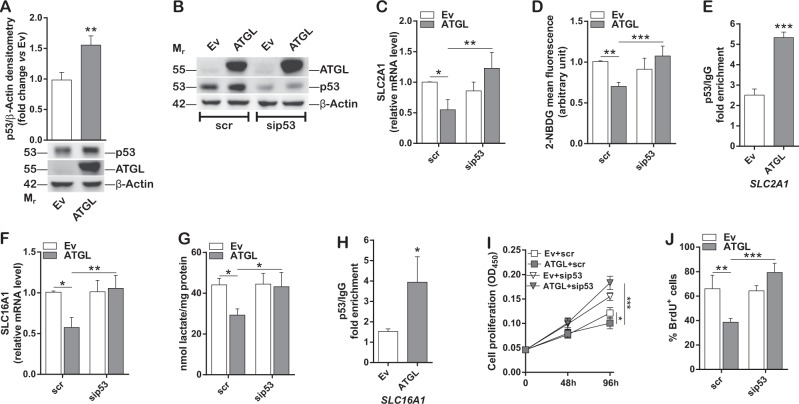
p53 is involved in ATGL-mediated metabolic adaptation and proliferation of HCC cell lines. HepG2 cells were transfected as previously described for 48 h. **A** Western blot analysis, representative of *n* = 6 independent experiments, of p53. β-Actin and ATGL were used as loading and transfection controls, respectively. Bar graph refers to the p53/β-Actin densitometry (^**^p < 0.01 *vs.* Ev). **B** HepG2 cells were silenced for p53 and overexpressed for ATGL as described in Materials and methods. Western blot analysis represents the p53 silencing control. β-Actin and ATGL were detected as loading and transfection controls, respectively. The image is representative of *n* = 3 independent experiments. **C** RT-qPCR analysis of *SLC2A1/GLUT1* and **F** of *SLC16A1/MCT1* expression upon p53 silencing. *ACTB* was used as reference control (*n* = 3; ^*^p < 0.05; ^**^p < 0.01). **D** Cytofluorimetric analysis of glucose uptake after p53 silencing. 10,000 events were counted (*n* = 3; ^**^p < 0.01; ***p < 0.001). **E** ChIP analysis performed on *SLC2A1/GLUT1* and **H** on *SLC16A1/MCT1* promoter regions. Data are expressed as fold enrichment and normalized on background signal (IgG) (*n* = 3; ^*^p < 0.05; ^***^p < 0.001 *vs.* Ev). **G** Extracellular lactate content was measured upon p53 silencing and ATGL overexpression; concentrations were normalized on total proteins (*n* = 6; ^*^p < 0.05). **I** Proliferation was assayed by CCK-8 colorimetric assay (*n* = 3; ^*^p < 0.05, ^***^p < 0.001) and **J** BrdU incorporation assay upon p53 silencing and ATGL overexpression. Bar graph refers to the percentage of BrdU-positive cells (*n* = 3; ^**^p < 0.01; ^***^p < 0.001)

Prompted by the observation that ATGL overexpression did not recapitulate the reduction of glycolysis and proliferation in Hep3B cells, which are p53 null, the role of this tumor suppressor as a possible effector of ATGL was investigated. Indeed, the negative regulation of glycolysis is one of the anti-neoplastic features of p53 [27, 28]. Interestingly, ATGL levels positively correlated with p53 protein levels (Fig. 4A), as also stated by ATGL silencing (Supplementary Figure S5A). In order to test the hypothesis of an ATGL-p53 functional axis, p53 was silenced upon ATGL overexpression (Fig. 4B) and glycolysis and proliferation examined. As reported in Fig. 4C, F, p53 silencing reverted the ATGL-mediated decreased expression of both *SLC2A1/GLUT1* and *SLC16A1/MCT1*, respectively, thus resulting in restored glucose uptake (Fig. 4D) and higher extracellular lactate concentration (Fig. 4G), with respect to the control. Interestingly, ChIP analyses revealed that the promoter region of both *SLC2A1/GLUT1* (Fig. 4E) and *SLC16A1/MCT1* (Fig. 4H) genes was bound to p53 to a higher extent in ATGL overexpressing cells than in the controls. Additionally, p53 silencing also resulted in proliferation recovery upon ATGL overexpression in HepG2 cells (Fig. 4I, J; Supplementary Figure S5B, C). Moreover, when p53 was transiently re-expressed in Hep3B cells (Supplementary Figure S5D), we observed a slight but not significant reduction of glycolysis (Supplementary Figure S5E–H) and proliferation (Supplementary Figure S5I–K), that was instead exacerbated upon ATGL overexpression.

It is worth mentioning that p53 directly controls mitochondrial activity mainly via regulation of the cytochrome c oxidase assembly factor SCO2 [29]. However, under our experimental conditions p53 induction by ATGL overexpression did not alter the levels of SCO2 in HepG2 cells (Supplementary Figure S5L). Moreover, p53 silencing did not revert the expression levels of the FA-metabolism related genes (Supplementary Figure S5M–Q), that we previously showed to be upregulated by ATGL.

### ATGL-mediated p53 acetylation by the PPAR-α/p300 axis is responsible for its inhibitory effect on glycolysis

**Fig. 5:**
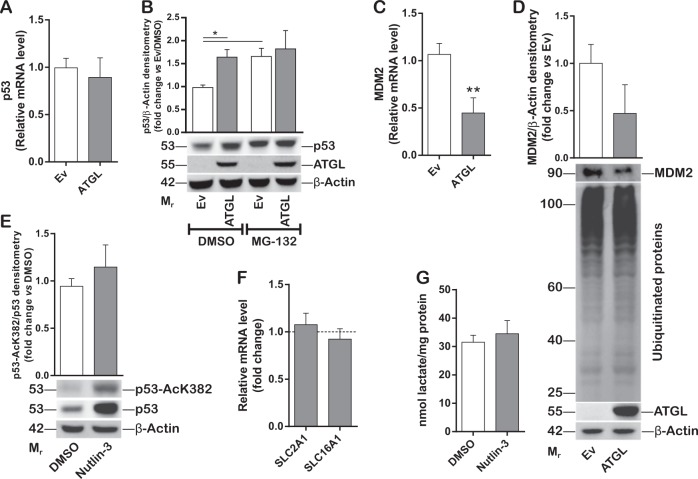
p53 accumulation is not sufficient to exert the effects observed downstream of ATGL. HepG2 cells were transfected as previously described. **A** RT-qPCR analysis of the expression levels of *TP53* (referred to as *p53*). *ACTB* was used as reference control (*n* = 6). **B** Cells were treated with 10 µM of the proteasome inhibitor MG-132 for 4 h and Western blot analysis performed to detect p53 levels. β-Actin and ATGL were used as loading and transfection controls, respectively. Bar graph refers to the p53/β-Actin densitometry (*n* = 3; ^*^p < 0.05). **C** RT-qPCR analysis of *MDM2*. *ACTB* was used as reference control (*n* = 6; ^**^p < 0.01 *vs.* Ev). **D** Western blot analysis of MDM2 and ubiquitinated proteins. β-Actin was used as loading control. ATGL was detected as transfection positive control. Bar graph refers to the MDM2/β-Actin densitometry analyses (*n* = 4). **E** HepG2 were treated with 1 µM of the MDM2 inhibitor Nutlin-3 for 24 h. Western blot analysis of p53 and of acetylated p53 (p53-AcK382). Bar graph refers to the densitometry p53-AcK382/p53 ratio. β-Actin was used as loading control (*n* = 3). **F** *SLC2A1/GLUT1* and *SLC16A1/MCT1* expression (*n* = 3) and **G** extracellular lactate content (*n* = 3) were evaluated upon Nutlin-3 treatment. Dashed line in **F** represents the negative control (DMSO)

The identification of p53 as downstream effector of ATGL prompted us to investigate the mechanism(s) by which ATGL induced p53 increase. ATGL overexpression augmented p53 protein (Fig. 4A), but not its mRNA (Fig. 5A) levels. Higher nuclear localization of p53 was disclosed upon ATGL overexpression (Supplementary Figure S6A, B).

Under normal conditions, p53 is unstable and one of the mechanisms controlling its levels is the rapid degradation by the ubiquitin/proteasome system [30]. In order to reveal whether ATGL interfered with this process, HepG2 cells were treated with the proteasome inhibitor MG-132 in combination with ATGL overexpression. Remarkably, no further statistically significant increase of p53 was observed upon proteasome inhibition in ATGL overexpressing cells, disclosing an ATGL-mediated protection of p53 from proteasomal degradation (Fig. 5B). No alteration of the total ubiquitinated protein profile was observed when ATGL was over-expressed (Fig. 5D). Interestingly, the expression levels of the E3 ubiquitin–protein ligase MDM2, the main negative regulator of p53 triggering its proteasomal degradation [30], were decreased both at mRNA and protein levels upon ATGL overexpression (Fig. 5C, D).

**Fig. 6:**
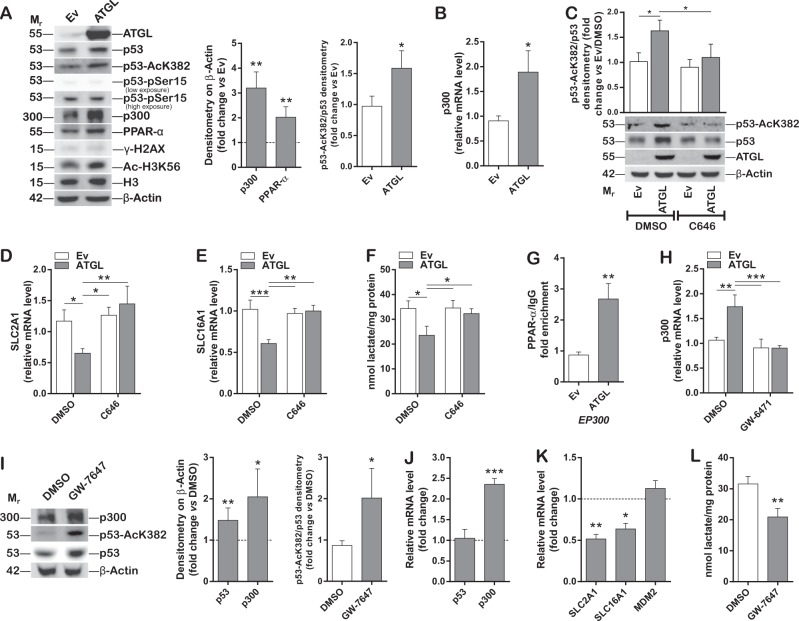
ATGL mediates p53 stabilization via acetylation by the PPAR-α/p300 axis. HepG2 cells were transfected as previously described. **A** Western blot analysis of p53, p53-AcK382, p53- pSer15, p300, PPAR-α, γ-H2AX, Ac-H3K56, and H3. β-Actin and ATGL were used as loading and transfection controls, respectively. Bar graphs refer to densitometry analyses (*n* = 4; ^*^p < 0.05; ^**^p < 0.01 *vs.* Ev, represented by a dashed line in the left bar graph). **B** RT-qPCR analysis of *EP300* (referred to as p300) expression. *ACTB* was used as reference control (*n* = 6; ^*^p < 0.05 *vs.* Ev). **C** HepG2 cells were treated with 10 µM of the p300 inhibitor C646 for 24 h in combination with ATGL overexpression. Western blot analysis of p53 and p53-AcK382. ATGL and β-Actin were used as transfection and loading control, respectively. Bar graph refers to densitometry p53-AcK382/p53 ratio (*n* = 3; ^*^p < 0.05). **D** *SLC2A1*/*GLUT1* and **E** *SLC16A1/MCT1* expression and **F** extracellular lactate content were evaluated upon C646 treatment and ATGL overexpression (*n* = 3; ^*^p < 0.05; ^**^p < 0.01; ^***^p < 0.001). **G** ChIP analysis performed on *EP300* promoter region to reveal PPAR-α interaction. Data are expressed as fold enrichment and normalized on background signal (IgG) (*n* = 3; ^**^p < 0.01 *vs.* Ev). **H** Cells were treated for 24 h with 5 µM of the PPAR-α antagonist GW-6471 and p300 expression determined by RT-qPCR (*n* = 3; ^**^p < 0.01; ^***^p < 0.001). **I** Representative Western blot (*n* = 3) of HepG2 cells treated for 24 h with 1 µM of the PPAR-α agonist GW-7647. Anti-p300, anti-p53-AcK382, and anti-p53 primary antibodies were used. β-Actin was used as loading control. Bar graphs refer to densitometry analyses (*n* = 4; ^*^p < 0.05; **p < 0.01 *vs.* DMSO, represented by a dashed line in the left graph). **J** RT-qPCR analysis of the expression levels of p53 and p300 and of **K** *SLC2A1*/*GLUT1*, *SLC16A1*/*MCT1*, and *MDM2* in HepG2 treated with GW-7647. *ACTB* was used as reference control. Data are displayed as fold change *vs.* DMSO, represented by a dashed line (*n* = 3; ^*^p < 0.05; ^**^p < 0.01; *vs.* DMSO). **L** Extracellular lactate content was evaluated upon GW-7647 treatment as previously described (*n* = 3; ^**^p < 0.01 *vs.* DMSO)

To assess whether diminished MDM2 expression could justify the effects of p53 accumulation observed downstream of ATGL, HepG2 cells were treated with the MDM2 inhibitor Nutlin-3. Although, as expected, Nutlin-3 treatment increased p53 protein levels (Fig. 5E), it did not alter either *SLC2A1*/*GLUT1* and *SLC16A1*/*MCT1* expression (Fig. 5F) or extracellular lactate content (Fig. 5G), suggesting that p53 increase is not sufficient to recapitulate the effects of the ATGL/p53 axis. Next, we investigated on additional mechanisms fostering p53 stabilization upon ATGL overexpression. p53 acetylation at Lys382 is an important post-translational modification responsible for its stabilization [31, 32]. Interestingly, ATGL overexpression increased the levels of the acetylated form of p53 at Lys382 (p53-AcK382) and the p53-AcK382/p53 ratio (Fig. 6A). Strikingly, the nuclear histone acetyltransferase p300, which is known to mediate this post-translational modification [32, 33], was upregulated (Fig. 6A, B), more active—as stated by increased acetylation of its target histone H3 at Lys56 (Ac-H3K56) (Fig. 6A)—and mainly localized in the nucleus (Supplementary Figure S6A) upon ATGL overexpression. Contrarily, ATGL silencing in HepG2 resulted in reduction of both p300 and of the p53-AcK382/p53 ratio (Supplementary Figure S5A). Moreover, the expression and the activity of the NAD^+^-dependent protein deacetylase sirtuin-1 (SIRT1), which is required for p53 deacetylation at Lys382 [34], were not affected upon ATGL overexpression (Supplementary Figure S6C, S6D). In order to confirm a functional role of p300 activity in both the acetylation of p53 and in the ATGL-mediated effects, p300 was inhibited by C646 in ATGL overexpressing HepG2 cells. As showed, diminished p53 acetylation (Fig. 6C), restored *SLC2A1*/*GLUT1* (Fig. 6D) and *SLC16A1*/*MCT1* (Fig. 6E) expression and extracellular lactate concentration (Fig. 6F) were depicted in these conditions.

In search of the missing link between ATGL and p300, the ATGL-downstream transcription factor PPAR-α was analyzed. Indeed, beyond a bioenergetic role, ATGL-released FAs can bind and transcriptionally activate PPAR-α [10, 12, 13]. Interestingly, PPAR-α protein levels, as well as nuclear localization, were increased upon ATGL overexpression (Fig. 6A; Supplementary Figure S6A). Coherently, PPAR-α protein levels were decreased when ATGL was silenced (Supplementary Figure S5A). Strikingly, ChIP analyses of ATGL overexpressing HepG2 nuclear extracts revealed that PPAR-α interacted with the promoter region of the p300 gene (*EP300*) to a higher extent than the control (Fig. 6G). The role of PPAR-α in the regulation of p300 expression upon ATGL overexpression was validated by the use of GW-6471, a specific PPAR-α antagonist (Fig. 6H; Supplementary Figure S6E). In line with these results, we found that the PPAR-α agonist GW-7647 recapitulated the events observed downstream of ATGL activity, including p300 upregulation and increased p53-AcK382/p53 ratio (Fig. 6I, J), decrement of *SLC2A1*/*GLUT1* and *SLC16A1*/*MCT1* expression (Fig. 6K) and diminished extracellular lactate content (Fig. 6L). These parameters were analyzed in cells co-treated with GW-7647 and C646 and confirmed the relevance of PPAR-α and p300 in mediating the signaling cascade (Supplementary Figure S7A–D). Moreover, no change in the expression levels of *MDM2* were depicted upon GW-7647, indicating that reduction of *MDM2* expression upon ATGL overexpression was not directly depending on PPAR-α signaling (Fig. 6K).

Hence, p53 acetylation—rather than its sole accumulation—is required to allow p53 to exert ATGL-triggered effects. Indeed, Nutlin-3 treatment did not alter the p53-AcK382/p53 ratio (Fig. 5E). To confirm this hypothesis, HepG2 were co-treated with Nutlin-3 and GW-7647, and no further decrement in *SLC2A1*/*GLUT1* (Supplementary Figure S7E) and *SLC16A1*/*MCT1* (Supplementary Figure S7F) expression nor in extracellular lactate content (Supplementary Figure S7G) were highlighted, with respect to GW-7647-treated cells.

### ATGL-mediated metabolic reprogramming confers susceptibility to glycolysis inhibitors.

**Fig. 7:**
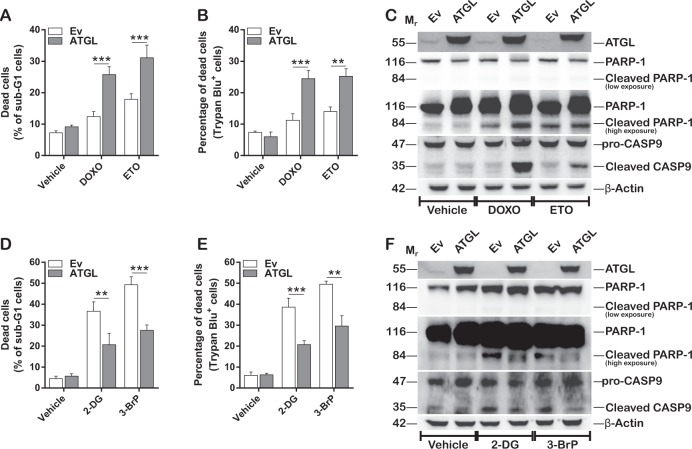
ATGL-mediated metabolic reprogramming confers susceptibility to glycolysis inhibitors. **A** HepG2 cells were transfected for ATGL for 48 h and treated with the genotoxic drugs doxorubicin (DOXO, 2 μM) and etoposide (ETO, 50 μM) for 16 h. The percentage of dead cells was assayed by propidium iodide and **B** Trypan blue procedures (*n* = 3; ^**^p < 0.01; ^***^p < 0.001). In **A**, the cytofluorimetric analysis was performed in FL-2 channel and 10,000 events were counted. The number of dead cells was expressed as percentage of sub-G1 cells. **C** The apoptotic cascade was analyzed by revealing the cleaved forms of CASP9 and PARP-1 by Western blot (*n* = 4); β-Actin was used as loading control. ATGL was detected as transfection positive control. **D** Cells were treated with the glycolysis inhibitors 2-deoxyglucose (2-DG, 30 mM) and 3-bromopyruvate (3-BrP, 30 μM) for 24 h upon ATGL overexpression. The percentage of dead cells was assayed by propidium iodide and **E** Trypan blue procedures (*n* = 3; ^**^p < 0.01; ^***^p < 0.001). **F** The apoptotic cascade was analyzed by revealing the cleaved forms of CASP9 and PARP-1 by Western blot (*n* = 3); β-Actin was used as loading control. ATGL was detected as transfection positive control

Finally, we investigated whether ATGL status conferred susceptibility to drug-induced cell death, which might provide useful hints for treatment of HCC. To this aim, HepG2 cells were challenged with 2 µM doxorubicin (DOXO) and 50 µM etoposide (ETO) for 16 h upon ATGL overexpression. First, we noted that ATGL overexpression alone was not able to induce apoptosis, despite it increased the levels of p53, which is a well-known apoptosis inducer [35]. This observation might be explained by the absence of induction of a DNA damage response, as no increase in the phosphorylation of histone H2AX at Ser139 (γ-H2AX) and of p53 at Ser15 (p53-pSer15) (Fig. 6A) was observed. Nevertheless, ATGL overexpression significantly increased cell death in response to both genotoxic drugs (Fig. 7A, B), together with the appearance of the cleaved forms of caspase 9 (CASP9) and poly(ADP-ribose) polymerase 1 (PARP-1), indicative of apoptosis (Fig. 7C).

Since we have demonstrated that ATGL overexpression redirect HCC cell metabolism towards a less glycolytic phenotype, we challenged ATGL overexpressing HepG2 cells with the glycolysis inhibitors 2-deoxyglucose (2-DG) and 3-bromopyruvate (3-BrP), at final concentrations of 30 mM and 30 µM, respectively, for 24 h. Our results demonstrated that in these conditions 2-DG and 3-BrP were less effective than the genotoxic drugs in inducing apoptosis upon ATGL overexpression (Fig. 7D–F).

These results indicate that ATGL protective function against cell death is specific for glycolysis-targeting agents and does not apply to all instances of cell death, thus strengthening the relevance of ATGL in liver metabolism and suggesting personalized therapeutic approaches in relation to ATGL expression.

## Discussion

HCC is the most common primary liver cancer type and its development is related to different risk factors, including chronic liver inflammation, cirrhosis, viral infections, and toxin exposure [1]. In the last years, changes in the dietary habits added new contributors to HCC development, among which is steatohepatitis [24]. Interestingly, ATGL, the main regulator of TGs hydrolysis in the liver [18, 23], was envisaged to be involved in the protection from steatohepatitis [21]. However, the contribution of ATGL to cancer and cancer-associated morbidities (*e.g.*, cachexia) is still lacking and controversial [16, 17, 36]. Indeed, albeit in vitro studies have proposed both pro-neoplastic [37, 38] and anti-neoplastic [39] roles for ATGL, the current in vivo observations indicate that ATGL is reduced in pancreatic adenocarcinoma [17] and in several other cancer types [16]. Indeed, ATGL loss is crucially implicated in the spontaneous development of pulmonary neoplasia [16] and of liposarcoma [40]. Driven by the evidence that ATGL orchestrates lipid signaling other than their homeostasis in the liver [18], possible implications of this lipase in liver cancer were investigated in our study. In line with data reported for other cancer types [16, 17], and with those provided by a publicly available microarray database, our findings reveal that ATGL transcript is remarkably reduced in human HCC, as well as in the diet-induced murine HCC model of steatohepatitis, in which immunohistochemical staining revealed a dramatic decrement of ATGL levels within cancerous lesions. Therefore, the molecular mechanisms affected by ATGL downregulation in HCC were explored. To this aim, ATGL was overexpressed in three different liver cancer cell lines (*i.e.*, HepG2, Huh7.5, and the p53-null Hep3B cells) and the main features were confirmed by ATGL silencing by RNA interference.

Our first observations revealed that ATGL levels inversely correlated with the proliferation rate of HCC-derived cell lines and this effect was dependent on intact ATGL enzymatic activity. In fact, the overexpression of a mutant ATGL lacking its catalytic activity failed to reduce cell proliferation. However, albeit ATGL depletion was recently demonstrated to confer a more aggressive phenotype to lung carcinoma cells [39], this was not observed in our conditions as ATGL had no effect on cell migration (data not showed). Since it is generally believed that FAs are necessary for cancer cells to build up membranes and enhance proliferation/growth, one might argue that ATGL-released FAs may contribute to a proliferative phenotype, rather than an anti-proliferative one. However, depending on cell context, there is also growing evidence speaking in favor of FAs antiproliferative effects. Indeed, it has been showed that oleic acid and dietary FAs, such as Omega 3 polyunsaturated FAs, are able to induce cell cycle arrest and apoptosis in HepG2 cells [41] and to decrease cancer cell growth [42–44]. Moreover, although it is generally accepted that cancer cells mainly rely on de novo lipogenesis to generate cellular lipids [45], we here show that ATGL overexpression does not induce any significant changes in the expression of FAs synthesis enzymes, thus excluding that ATGL effects on proliferation involve an anabolic contribution. Recently, ATGL deficiency was also proposed to promote increased proliferation in a ROS-mediated fashion in different cell lines [46], but this was not observed in our model, thus arguing for alternative mechanisms contributing to the control of proliferation.

Noticeably, high-proliferation rate of most cancer cells is a consequence of a metabolic reprogramming leading to an oxidative-to-glycolytic swap (the Warburg effect) which ensures the replenishment of fast glucose-derived energy and of substrates for anabolic reactions [3]. Therefore, we wondered if ATGL played a role in metabolic rewiring, given its important metabolic functions in the liver. We found that ATGL overexpression reduced LDs accumulation—which was reverted by ATGL silencing—in line with the massive lipid accumulation typical of ATGL-deficient mouse organs [10, 11]. Indeed ATGL widely impacted mitochondrial respiration: we observed increased mitochondrial uptake of FAs and higher lipids utilization, upregulation of Krebs cycle enzymes—which are commonly deregulated or mutated in many cancer types [47]—and higher respiratory rate, coupled with augmented ATP production, overall revealing an increased mitochondrial activity. Instead, no effect on either mitochondrial content—the main way by which mitochondria maximize their capacity—or the expression of the electron transport chain subunits, were detected. The effects on mitochondrial metabolism were observed in all three cell lines upon ATGL overexpression. In parallel, we examined the effects of ATGL on glycolysis: lower glucose uptake, extracellular lactate concentration, and decreased expression of *SLC2A1/GLUT1* and *SLC16A1/MCT1*, the glucose and lactate transporters commonly found altered in different types of cancer [48, 49], were remarked. All these events were dependent on intact ATGL enzymatic activity and reverted by ATGL silencing. However, only a barely increase in the expression of glucose and lactate transporters was observed upon ATGL silencing. This observation might be explained by the concept that ATGL levels are so low in HCC cells that its silencing does not trigger dramatic effects on gene expression. Another possibility is that some specific effects require the presence of FAs, that in the context of ATGL silencing are not efficiently released. Additionally, a reduction in the hexokinase-2 (HK-2) activity, the first check-point enzyme regulating the glycolytic flux [50], was observed. Notably, no change in the protein levels of HK-2, the main isoform expressed in cancer [50], was detected, suggesting possible post-translational modification(s) of the enzyme that were not investigated. Altogether, these findings unveil a crucial role for ATGL in the control of metabolic rewiring of HCC cell lines.

Strikingly, we noted partial phenotype overlapping between Hep3B and HepG2/Huh7.5 cells upon ATGL overexpression. Indeed, despite a higher oxidative phenotype in all cell lines, no ATGL effect on both proliferation and glucose metabolism was observed in Hep3B cells. Such variability was likely attributed to the functions of the tumor-suppressor p53, for which Hep3B cells are null. Indeed, p53 has a well-established role in the modulation of tumor metabolism, both as negative regulator of glycolysis and as inducer of mitochondrial respiration. p53 effects on glycolysis is exerted either by positive expression of the *TP53-*inducible glycolysis and apoptosis regulator (TIGAR) [51], or by negative regulation of glucose and lactate transporters [27, 28]. To drive further interests toward p53 was our finding of an increase of its protein levels upon ATGL overexpression, which was abrogated by ATGL silencing and unrelated to changes in p53 transcript. Therefore, p53 was silenced in ATGL overexpressing HepG2 cells, resulting in recovered glycolytic phenotype and proliferation. This effect was ascribed to the inhibitory role of p53 on *SLC2A1/GLUT1* and *SLC16A1/MCT1* expression by direct interaction with their promoter region, as active consequence of its increased nuclear localization, rather than activation of TIGAR, whose transcript was not altered by ATGL overexpression (data not showed). However, silencing of p53 alone had limited or no influence on proliferation/Warburg effect, likely arguing for only a minor role of p53 on such phenomena. However, it is widely accepted that p53 is kept under tight regulation in normal growth conditions [52]. For instance, Boidot et al. [27] proved that p53 represses SLC16A1/MCT1 expression only under hypoxic conditions in HCT116 colon cancer cells. In fact, SLC16A1/MCT1 protein levels are unvaried in WT *vs*. p53^−/−^ HCT116 cells under normoxia. On the other hand, p53 silencing is sufficient to increase lactate levels in human primary fibroblasts, compared to WT cells [53]. Therefore, our results may be justified by the absence of any specific stimuli impinging on p53 under basal conditions. The role of p53 as downstream effector of ATGL was also recapitulated in Hep3B cells, in which p53 was transiently re-expressed. Here, p53 re-expression impaired glycolysis and reduced proliferation, being these effects exacerbated upon ATGL overexpression. Interestingly, p53 can also affect oxidative metabolism, mainly by the activation of mitochondrial FAs uptake and β-oxidation genes and synthesis of SCO2 [29]. However, no implication of p53 in the oxidative metabolism was assessed in our model. Indeed, ATGL overexpression did not alter SCO2 expression in HepG2, while Hep3B cells showed a higher oxidative metabolism in the same conditions. Moreover, despite a barely increase was observed, p53 silencing did not significantly influence the expression of FA oxidation-related genes in combination with ATGL overexpression in HepG2 cells.

Considerably, the mechanism(s) implicated in p53 accumulation were investigated. Our analyses revealed that ATGL protected p53 from proteasomal degradation and that the expression levels of MDM2, the E3 ubiquitin–protein ligase driving p53 to proteasomal degradation [30], were remarkably decreased upon ATGL overexpression. In order to address whether p53 accumulation was sufficient to mediate the antiglycolytic effects observed downstream of ATGL, MDM2 was inhibited by Nutlin-3, but this did not affect either *SLC2A1/GLUT1* and *SLC16A1/MCT1* expression or extracellular lactate concentration, thus highlighting possible alternative mechanisms involved in p53 activation downstream of ATGL. It is well-established that ATGL-released FAs not uniquely increase oxidable substrates, but also bind and activate PPAR-α [10, 12, 13]. Indeed, protein levels of PPAR-α were increased upon ATGL overexpression and decreased by ATGL silencing. ATGL also induced higher PPAR-α nuclear localization compared to the control. Being FAs the primary carbon source for protein acetylation in hepatocytes [54], p53 acetylation, which is known to increase protein stability and activity [31, 32], was examined. Remarkably, ATGL levels positively correlated with p53 acetylation at Lys382 (p53-AcK382), as stated by both ATGL overexpression and silencing, and with the p53-AcK382/p53 ratio, which was not affected by Nutlin-3 exposure. Importantly, p53 acetylation was described to occur in a PPAR-α dependent fashion, as demonstrated by the use of the PPAR-α specific agonist GW-7647. Notably, the expression levels of MDM2 were not affected by GW-7647 treatment, indicating that its downregulation was not ascribable to the ATGL/PPAR-α signaling. Additionally, no further repression of glycolysis was observed upon GW-7647 and Nutlin-3 co-treatment, again devaluing a causative involvement of the MDM2-mediated p53 accumulation in the observed phenotype. Moreover, GW-7647 recapitulated the alterations in the glycolytic phenotype, speaking in favor of a crucial role for PPAR-α in effecting the ATGL/FAs signaling driving the metabolic rewiring of HCC cell lines.

In search of the missing link between PPAR-α activation and p53 acetylation/activation, the involvement of the nuclear histone acetyltransferase p300, the main player in p53 acetylation and whose inhibition prevents p53 activity [32, 33, 55], was inspected. Indeed, we found that p300 was up-regulated upon ATGL overexpression, being this reverted by ATGL silencing, and active, as stated by increased levels of Ac-H3K56. Interestingly, p300-mediated acetylation of H3 at Lys56 was reported to inhibit cell proliferation, colony formation and migration of cancer cell lines, including hepatoma, through regulation of gene expression [56]. Our data show an increase of Ac-H3K56 levels upon ATGL overexpression that may likely contribute to the antiproliferative effect observed in these conditions. p300 upregulation was traced back to PPAR-α activation, as revealed by higher interaction of PPAR-α with the *EP300* promoter region upon ATGL overexpression. This was confirmed both upon PPAR-α inhibition by GW-6471 in combination with ATGL overexpression and upon GW-7647 treatment. The involvement of p300 was also corroborated by selective inhibition of its acetyltransferase activity by C646, in combination with ATGL overexpression or GW-7647 treatment. Notably, though the activity of SIRT1, which is required for p53 deacetylation at Lys382 [34], was showed to be regulated by ATGL in different cellular processes [23, 57], no alterations of its levels or activity were disclosed in our model. Altogether, these findings give clear-cut evidence for a novel ATGL/PPAR-α/p300 axis involved in p53 acetylation, stabilization and activation in HCC cell lines, responsible for enhanced mitochondrial activity and proliferation. These results support the understanding of how ATGL downregulation in HCC participates in the maintenance of a glycolytic phenotype.

New therapeutic approaches targeting glucose addiction have emerged to face cancer progression [58], with the aim of limiting cell growth and triggering cell death [59, 60]. In this work, we observed that HCC cells treated with the glycolysis inhibitors 2-DG and 3-BrP were prone to apoptotic cell death, and this was reversed by ATGL overexpression, thus confirming the prominent role of ATGL in modulating metabolic rewiring in HCC. Contrarily, cells exposed to DNA damaging agents (*i.e.*, doxorubicin and etoposide) displayed increased cell death by apoptosis in presence of ATGL overexpression. These results unveiled a new potential role in cell death induction in response to genotoxic drugs exposure, although the mechanisms are still unknown. It is worth mentioning that acetylation of p53 at Lys382 is also required for triggering cell death [33, 61]. However, it is well accepted that p53 increase is not sufficient to induce cell death in all circumstances. This may partially explain why compounds targeting p53-MDM2 interaction (*e.g*. Nutlin-3) show low efficacy in clinical trial, as they fail to induce p53-dependent apoptosis in cancer cells [62]. Indeed, p53 triggers apoptosis in cells that are challenged by diverse types of stimuli, mainly oxidative stress, DNA damage and hypoxia. This was not observed in our conditions, where ATGL overexpression did not affect ROS production (data not showed), hypoxia (data not showed) or phosphorylation of both histone H2AX at Ser139 (γ-H2AX), the most sensitive marker used to determine the presence of DNA damage [63], and of p53 at Ser15, whose phosphorylation positively regulates apoptosis [35]. However, we don’t exclude the possibility that p53 could be involved in such phenomena. Work is still ongoing in our laboratory to fill this gap.

Comprehensively, in this work, we provide evidence for ATGL downregulation in both human and murine HCC and we describe the mechanisms whereby this lipase contributes to metabolic rewiring through p53 stabilization and the identification of a previously uncovered ATGL/PPAR-α/p300 axis. Moreover, we also identified a higher susceptibility to glycolysis-inhibiting insults of cells with reduced levels of ATGL. Overall, we herein propose a new tumor-suppressor function of ATGL in HCC, which might be exploited for creating personalized therapies, possibly in combination with existing therapeutic regimens, aimed at selectively kill cancer cells.

## Materials and methods

Diethylnitrosamine (DEN), dimethylsulfoxide (DMSO), paraformaldehyde, formaldehyde, glycine, TRI Reagent, Bromodeoxyuridine (BrdU), 2-deoxyglucose (2-DG), 3-bromopyruvate (3-BrP), doxorubicin (DOXO), etoposide (ETO), MG-132, C646, Nutlin-3, Oil Red-O (ORO), Propidium Iodide (PI), Triton X-100, Nonidet P-40 (NP-40), Adenosine 5′-triphosphate sodium salt (ATP), glucose, nicotinamide adenine dinucleotide phosphate sodium salt (NADP^+^), nicotinamide adenine dinucleotide (NAD^+^), and glucose-6-phosphate dehydrogenase were from Sigma-Aldrich. 2-(*N*-(7-Nitrobenz-2-oxa-1,3-diazol-4-yl)Amino)-2-Deoxyglucose (2-NBDG), MitoTracker^™^ Green FM (MTG), Hoechst 33342 and Alexa Fluor™ 568 donkey anti-mouse IgG (H + L) were from Thermo Fisher Scientific. Trypan blue 0.4% solution was from Lonza. (H + L)-horseradish peroxidase conjugated goat anti-mouse and anti-rabbit IgG were from Bio-Rad Laboratories. l-lactate dehydrogenase (LDH) was from Roche Applied Science. Polyethylenimine (PEI) was from Polysciences. GW-7647 and GW-6471 were from Cayman Chemical. Choline deficient diet (CDD) was from Research Diets.

Human HCC samples were kindly provided by Prof. Grazi from the Hepato-Pancreato-Biliary Surgery Unit, Department of Clinical and Experimental Oncology, Regina Elena National Cancer Institute, Rome, Italy. Analyses were performed after approval from the Regina Elena Cancer Institute ethical committee. All mouse experimentation was performed in accordance with accepted standard of human animal care and after approval by the Italian Ministry of Welfare committee and the Institutional Animal Care and Use Committee of the University of Rome “Tor Vergata” (Italy). C57BL/6 mice were purchased from Harlan Laboratories Srl (Urbino, Italy) and randomly divided in two groups; treated group (*n* = 7, male) was intraperitoneally injected with a 20 mg/kg DEN dose at 15 days after birth and fed a CDD at weaning. Reference group (*n* = 7, male) was intraperitoneally injected with saline solution and fed standard pellet, at weaning (ND). Hepatic status was periodically followed-up by analyses on hepatic clinical markers aspartate aminotransferase (GOT-AST) and alanine aminotransferase (GPT-ALT) on sera from retro orbital blood collection. Before sacrifice, mice were fasted for 4 h and euthanized. Livers were perfused with saline solution prior to all the analyses performed. Mice liver thick sections were also stained for H&E morphological evaluation and for ATGL expression by IHC.

For this study, three different liver cancer cell lines were used. HepG2 and Hep3B cell lines were purchased from Leibniz-Institut DSMZ, Braunschweig (Germany). Huh7.5 cell line was a kind gift from Dr. Carla Montesano, University of Rome “Tor Vergata” (Italy). All cell lines were grown in Dulbecco’s modified Eagle’s medium (DMEM) 1 g/L glucose supplemented with 10% fetal bovine serum, 2 mM l-glutamine, 10 U/ml penicillin/streptomycin and 1% MycoZap^™^. Cells were cultured at 37 °C in a 5% CO_2_ atmosphere and plated at a 2 × 10^5^ cells/ml density for all the experiments. After 24 h plating, cells were transiently transfected with pcDNA™4/HisMaxC and pcDNA™4/HisMaxC-ATGL or pEGFP, p*ATGL*^*WT*^-EGFP and p*ATGL*^*(Ser47Ala)*^-EGFP or pcDNA3.1 and pcDNA3.1-p53^WT^ plasmids, for the times indicated in the experiments, by the PEI reagent, according to manufacturer’s instructions. sip53 and siATGL were reversely transfected using Lipofectamine^®^ RNAiMAX Transfection Reagent (Thermo Fisher Scientific), according to the manufacturer’s protocol. GW-7647 and Nutlin-3 were used at a final concentration of 1 µM for 24 h. GW-6471 was used at a final concentration of 5 µM for 24 h. C646 and MG-132 were used at a final concentration of 10 µM for 24 h and 4 h, respectively. 2-DG and 3-BrP were used for 24 h at 30 mM and 30 µM final concentrations, respectively. DOXO and ETO were used for 16 h at 2 µM and 50 µM final concentrations, respectively.

TGs content was evaluated by Triglyceride Quantification Colorimetric/Fluorometric Kit (BioVision) and total neutral lipids stored in LDs by Oil Red-O staining procedure. Briefly, cells were fixed, rinsed in 60% isopropanol and stained with the Oil Red-O working solution. Oil Red-O was eluted and relative absorbance measured at 510 nm. Oxidative metabolism was examined by RT-qPCR analysis of both mitochondrial FAs uptake and β-oxidation genes and Krebs cycle genes, also by Western blot. FAs utilization was assessed by Seahorse Bioscience XF^e^96 analyzer in combination with the Seahorse XF Palmitate-BSA FAO Substrate assay kit. Mitochondrial function was assessed both using a Seahorse Bioscience XF^e^96 analyzer in combination with the Seahorse Bioscience XF Cell Mito Stress Test assay kit and by a Clark-type oxygen electrode. ATP levels were detected using the ATP Bioluminescence Assay Kit CLS II (Roche Applied Science). SIRT1 activity was determined by the SIRT1/Sir2 Deacetylase Fluorometric (Human) Assay Kit (Abnova).

Glycolysis was monitored by RT-qPCR analysis of *SLC2A1/GLUT1* and *SLC2A2/GLUT2* glucose transporters and *SLC16A1/MCT1* lactate transporter. Glucose uptake was cytofluorimetrically recorded in FL-1 channel by a FACScalibur instrument after a 1-h-incubation with 100 µM 2-NBDG. HK activity and extracellular lactate measurements were determined spectrophotometrically.

Proliferation was evaluated by Trypan blue staining, CCK-8 colorimetric assay (Cell Counting Kit-8, Dojindo Molecular Technologies) and by BrdU incorporation assay. For BrdU incorporation assay, cells were fixed and incubated with an anti-BrdU primary antibody (after DNA denaturization) and nuclei stained with 1 µg/ml Hoechst 33342. Images were digitized with a Delta Vision Restoration Microscopy System (Applied Precision) equipped with an Olympus IX70fluorescence microscope (Olympus Italia).

Nuclear fractions were obtained as previously described [64] and used for both Western blot and ChIP assays.

Cell viability was determined by Trypan Blue and Propidium Iodide staining procedures (dead cells were expressed as percentage of subG1 cells).

Data were presented as means ± SD. Statistical analyses were performed by unpaired 2-tailed Student *t* test and one- or two-way ANOVA. Comparisons were statistically considered significant at *p* ≤ 0.05 (*).

RT-qPCR, Western blot, and fluorescence microscopy procedures as well as complete and detailed description of all methods employed are reported in Supplementary information.

## Electronic supplementary material

Supplementary Materials

Supplementary Figure 1

Supplementary Figure 2

Supplementary Figure 3

Supplementary Figure 4

Supplementary Figure 5

Supplementary Figure 6

Supplementary Figure 7
