# Supplementary material for: Forcing ATGL expression in hepatocarcinoma cells imposes glycolytic rewiring through PPAR-α/p300-mediated acetylation of p53
Source: Oncogene. 2018 Oct 26;38(11):1860–75. doi: 10.1038/s41388-018-0545-0 (PMC6756110; doi:10.1038/s41388-018-0545-0)
Supplement: Supplementary file 1 — Supplementary Materials [file 41388_2018_545_MOESM1_ESM.docx]

# Supplementary Figure Legends

***Figure S1.*** (**A**) Bioclinical analyses for the determination of the hepatic status of mice groups on sera obtained from retro orbital blood collection. The hepatic markers GOT-AST (**B**) and GPT-ALT were analyzed. Grey sections on graphs represent normal value ranges (n=14; ** *p<0.01* CDD+DEN *vs* ND). (**C**) RT-qPCR analysis of the hepatic tumor marker *Afp* in tumor biopsies (T) from mice treated with CDD+DEN *vs* respective non-tumor counterparts (NT). *Actb* was used as reference control (n=14; *** *p<0.001 vs* NT).

***Figure S2.*** (**A**) HepG2 cells were transfected for ATGL for 48h and 96h to determine ATGL transient over-expression duration. β-Actin was used as loading control (n=3). (**B**) HepG2 cells were transfected for ATGL for the times indicated and proliferation assayed by Trypan blue direct cell counting procedure (n=3; ** *p<0.01 vs* Ev). (**C**) HepG2 cells were silenced for ATGL for 96h and proliferation assayed by BrdU incorporation assay. The bar graph and the Western blot refer to BrdU positive cells and to the silencing control, for which β-Actin was used as loading control, respectively (n=3; ** *p<0.01 vs* scr). (**D**) HepG2 cells were transfected with p*ATGLWT*-EGFP, p*ATGL(Ser47Ala)*-EGFP and pEGFP plasmids; after 96h from transient transfection, ATGL expression levels were detected by Western blot, using β-Actin as loading control and (**E**) proliferation determined by direct cell counting according to the Trypan blue staining procedure (n=3; * *p<0.05*;

** *p<0.01*). (**F**) Huh7.5 and Hep3B cells were transfected for ATGL and proliferation respectively assayed in both cells lines at 96h by (**G**, **J**) CCK-8 colorimetric assay (n=6; * *p<0.05 vs* Ev), (**H**, **K**) Trypan blue direct cell counting procedure (n=3; ** *p<0.01 vs* Ev) and (**I**, **L**) BrdU incorporation assay. Images reported in (**I**) and (**L**) are representative of n=3 independent experiments. Bar graphs refer to the percentage of BrdU positive cells (n=3; *** *p<0.001 vs* Ev).

***Figure S3.*** (**A**) HepG2 cells were silenced for ATGL for 48h and LDs content measured according to the Oil Red-O staining procedure (n=3; * *p<0.05 vs* scr). (**B**) RT-qPCR analysis of the expression levels of lipid biogenesis genes (*ACC1*, *FASN*). *ACTB* was used as reference control. Data are showed as fold change *vs* Ev, represented by a dashed line (n=6). (**C**) Basal and (**D**) maximal respiration, (**E**) spare respiratory capacity, (**F**) non-mitochondrial oxygen consumption, (**G**) proton leak and (**H**) coupling efficiency were determined in ATGL over-expressing HepG2 cells by the Seahorse Bioscience XFe96 analyzer in combination with the Seahorse Bioscience XF Cell Mito Stress Test assay kit (n=3; * *p<0.05*; ** *p<0.01*; *** *p<0.001 vs* Ev). (**I**) HepG2 mitochondrial content was assessed by both Western blot (n=3) analysis on the mitochondrial markers PGC-1α, HSP60 and TOMM20, using β-Actin as loading control, and (**J**) by cytofluorimetric determining MTG fluorescence in FL-1 channel (10,000 events were counted) (n=3) upon ATGL over-expression (48h). (**K**) RT-qPCR analysis of the expression levels of nuclear and (**L**) of mitochondrial encoded electron transport chain genes, depicted also by Western blot, as reported in (**I**). Data in (**K**) and (**L**) are showed as in (**B**) (n=3). (**M**) LDs content was spectrophotometrically measured by Oil Red-O staining procedure in both Huh7.5 and (**Q**) Hep3B cells upon ATGL over-expression (n=3; ** *p<0.01 vs* Ev). (**N**) RT-qPCR analysis of mitochondrial FAs uptake (*Cpt1A*, *Cpt2*) and mitochondrial β- oxidation (*ACADM*, *ACADS*) genes in Huh7.5 and (**R**) Hep3B cell lines upon ATGL over-expression. *ACTB* was used as reference control. Data are showed as in (**B**) (n=6; * *p<0.05*; ** *p<0.01 vs* Ev).

(**O**) RT-qPCR analysis of Krebs cycle genes in Huh7.5 and (**S**) Hep3B cells upon ATGL over- expression. *ACTB* was used as reference control. Data are graphed as in (**B**) (n=6; * *p<0.05*; ** *p<0.01 vs* Ev). (**P**) Oxidative phosphorylation was monitored by measuring ATP content by

luminometric assays upon ATGL over-expression in Huh7.5 and (**T**) Hep3B cell line (n=3; * *p<0.05*;

** *p<0.01 vs* Ev).

***Figure S4.*** (**A**) HepG2 cells were silenced for ATGL for 48h and *SLC16A1/MCT1* and *SLC2A1/GLUT1* expression and (**B**) extracellular lactate content measured (n=3; ** *p<0.01 vs* scr). In (**A**), data are showed as fold change vs scr, represented by a dashed line. (**C**) RT-qPCR analysis of *SLC16A1/MCT1*, *SLC2A1/GLUT1* and *SLC2A2/GLUT2* in Huh7.5 and (**F**) Hep3B cells upon ATGL over- expression. *ACTB* was used as reference control. Data are showed as fold change *vs* Ev, represented by a dashed line (n=6; ** *p<0.01*; *** *p<0.001 vs* Ev). (**D**) Cytofluorimetric analysis in FL-1 channel of glucose uptake in Huh7.5 and (**G**) Hep3B cells upon ATGL over-expression. 10,000 events were considered (n=3; ** *p<0.01 vs* Ev). (**E**) Extracellular lactate content was measured in Huh7.5 and (**H**) Hep3B cells. Concentrations were normalized on total proteins (n=3, ** *p<0.01 vs* Ev). (**I**) ATGL activity was spectrophotometrically determined 48h after p*ATGLWT*-EGFP, p*ATGL(Ser47Ala)*-EGFP and pEGFP plasmids over-expression by Oil Red-O staining procedure (n=4; ** *p<0.01 vs* pEGFP).

***Figure S5.*** (**A**) HepG2 cells were silenced for ATGL for 48h and p53, p53-AcK382, p300 and PPAR-α levels detected by Western blot. β-Actin and ATGL were used as loading and silencing controls, respectively. Bar graphs refer to densitometry analyses (n=3; * *p<0.05*; ** *p<0.01*; *** *p<0.001 vs* scr, represented by a dashed line in the left bar graph). (**B**) HepG2 cells were silenced for p53 and over-expressed for ATGL as described in *Materials and Methods*. Proliferation was assayed by BrdU incorporation assay (images reported are representative of n=3 independent experiments) and (**C**) by direct cell counting by Trypan blue staining procedure (n=3; * *p<0.05*, *** *p<0.001*). (**D**) Hep3B cells were co-transfected for ATGL and p53 (with a pcDNA3.1 and a pcDNA3.1-p53WT plasmid) for 48h. Western blot was performed to check p53 expression. ATGL and β-Actin were used as transfection and loading control, respectively. (**E**) In these cells, RT-qPCR analysis of *SLC2A1/GLUT1* and (**G**) of *SLC16A1/MCT1* were performed. *ACTB* was used as reference control. Data are showed as fold change *vs* Ev+pcDNA3.1 (n=3; * *p<0.05*, ** *p<0.01*, *** *p<0.001*). (**F**) Glucose uptake was cytofluorimetrically assessed by 2-NBDG incorporation in ATGL and p53 co- transfected Hep3B cells. 10,000 events were counted and data expressed as arbitrary units (n=3; * *p<0.05*; *** *p<0.001*). (**H**) Extracellular lactate content was measured as previously described upon ATGL and p53 co-transfection in Hep3B cells. Concentrations were normalized on total proteins (n=3; ** *p<0.01*; *** *p<0.001*). (**I**) Hep3B cells were co-over-expressed for ATGL and p53 for 48h and 96h and BrdU incorporation assay, (**J**) CCK-8 colorimetric assay and (**K**) direct cell counting by Trypan blue staining procedure performed (n=3; * *p<0.05*; ** *p<0.01*). Images in (**I**) are representative of n=3 independent experiments. Bar graph refers to the percentage of BrdU positive cells (* *p<0.05*, *** *p<0.001*). (**L**) HepG2 cells were over-expressed for ATGL for 48h and expression levels of *SCO2* determined by RT-qPCR, using *ACTB* as reference control. (n=3). (**M**) HepG2 were silenced for p53 upon ATGL over-expression and *Cpt1A*, (**N**) *Cpt2*, (**O**) *ACADM*, (**P**) *ACADS* and (**Q**) *CS* -citrate synthase- detected by RT-qPCR. *ACTB* was used as reference control. Data are showed as fold change *vs* scr (n=3; * *p<0.05*; ** *p<0.01*).

***Figure S6.*** (**A**) HepG2 were over-expressed for ATGL for 48h and nuclear fraction isolated. Western blot analysis, representative of n=3 independent experiments, was carried out to detect p300, p53, and PPAR-α levels in nuclear fraction. LDH and Lamin B1 were used as purity and loading controls for the post-nuclear and nuclear fractions, respectively. (**B**) Immunofluorescent analysis of p53 subcellular localization in HepG2 cells after ATGL over-expression. Images are

representative of n=3 independent experiments. Nuclei were stained with 1 µg/ml Hoechst 33342.

(**C**) SIRT1 activity was determined fluorometrically and normalized on proteins. The Western blot refers to the protein levels of SIRT1 upon ATGL over-expression (n=3). β-Actin was used as loading control (n=3). (**D**) *SIRT1* expression levels were determined by RT-qPCR upon ATGL over- expression. *ACTB* was used as reference control (n=3). (**E**) Cells were treated for 24h with 5µM of the PPAR-α antagonist GW-6471 upon ATGL over-expression and p300 expression determined by Western blot. β-Actin was used as loading control. (n=3).

***Figure S7.*** (**A**) HepG2 cells were co-treated with 10µM C646 and 1µM GW-7647 for 24h and Western blot for p53 and p53-AcK382 performed. β-Actin was used as loading control. Bar graph refers to the p53-AcK382/p53 densitometry ratio (n=3; * *p<0.05*; ** *p<0.01*). (**B**) *SLC2A1/GLUT1*,

(**C**) *SLC16A1/MCT1* expression and (**D**) extracellular lactate content were also evaluated (n=3; * *p<0.05*; ** *p<0.01*; *** *p<0.001*). (**E**) In all the experiments, HepG2 cells were treated with 1µM Nutlin-3 and 1µM GW-7647 in combination or not for 24h. *SLC2A1/GLUT1* and (**B**) *SLC16A1/MCT1* expression and (**C**) extracellular lactate content were analyzed as previously described (n=3; * *p<0.05*; ** *p<0.01*).

# Supplementary Materials and Methods

*Materials.*

Diethylnitrosamine (DEN), dimethylsulfoxide (DMSO), paraformaldehyde, formaldehyde, glycine, TRI Reagent, Bromodeoxyuridine (BrdU), 2-deoxyglucose (2-DG), 3-bromopyruvate (3-BrP), doxorubicin (DOXO), etoposide (ETO), MG-132, C646, Nutlin-3, Oil Red-O (ORO), Propidium Iodide (PI), Triton X-100, Nonidet P-40 (NP-40), Adenosine 5′-triphosphate sodium salt (ATP), glucose, nicotinamide adenine dinucleotide phosphate sodium salt (NADP+), nicotinamide adenine dinucleotide (NAD+) and glucose-6-phosphate dehydrogenase were from Sigma-Aldrich. 2-(*N*-(7- Nitrobenz-2-oxa-1,3-diazol-4-yl)Amino)-2-Deoxyglucose (2-NBDG), MitoTracker™ Green FM (MTG), Hoechst 33342 and Alexa Fluor™ 568 donkey anti-mouse IgG (H+L) were from Thermo Fisher Scientific. Trypan blue 0.4% solution was from Lonza. (H+L)-horseradish peroxidase conjugated goat anti-mouse and anti-rabbit IgG were from Bio-Rad Laboratories. L-lactate dehydrogenase (LDH) was from Roche Applied Science. Polyethylenimine (PEI) was from Polysciences. GW-7647 and GW-6471 were from Cayman Chemical. Choline Deficient Diet (CDD) was from Research Diets.

*Animals.*

All mouse experimentation was performed in accordance with accepted standard of human animal care and after approval by the Italian Ministry of Welfare committee and the Institutional Animal Care and Use Committee of the University of Rome “Tor Vergata”, Italy. C57BL/6 mice were purchased from Harlan Laboratories Srl (Urbino, Italy). Hepatocellular carcinoma was obtained in mice as previously described 3. Male mice came from different litters and were randomly divided in order to obtain homogeneous groups in number. The minimal number of mice was chosen in accordance with the current Italian rules on mouse experimentation and to ensure the adequate statistical power to our analyses (minimum 6 animals per group). At 15 days after birth, mice were intraperitoneally injected with a unique 20mg/kg dose of the hepatotoxic compound Diethylnitrosamine (DEN) and fed *ad libitum* with a Choline Deficient Diet (CDD) (n=7), responsible for hepatosteatosis development 2, at weaning. Reference group was intraperitoneally injected with saline solution and fed *ad libitum* with standard pellet, at weaning (Normal Diet, ND) (n=7). All the animals had free access to water and were treated for 45 weeks. Hepatic status of mice groups was periodically and blinding followed-up by analyses conducted on the hepatic clinical markers aspartate aminotransferase (GOT-AST) and alanine aminotransferase (GPT-ALT) on sera obtained from retro orbital blood collection, with the valid collaboration of Dr. Roberta Bernardini at the Centro di servizi interdipartimentale - Stazione per la tecnologia animale, University of Rome “Tor Vergata”, Italy. All the CDD+DEN mice developed HCC at the end of the experimentation and were all included in the study. Before sacrifice, mice were fasted for 4h and euthanized. Livers were perfused with saline solution, immediately explanted and processed or, alternatively, frozen in liquid nitrogen and stored at -80 °C.

*H&E staining and Immunohistochemistry.*

After fixation in 10% buffered formalin for 24h, mouse liver tissues were embedded in paraffin. Three-micrometre thick sections were stained with haematoxylin and eosin (H&E). Hepatic parenchyma alterations, morphology and necrosis were blindly evaluated by two pathologists. The

reference range for the parameters analysed was 0-3, where 0 represented a “null condition” and 3 a “maximum condition”. Immunohistochemical analysis was performed to assess the expression of ATGL in mice liver sections. Briefly, four-micrometre thick sections from paraffin blocks were pre-treated with EDTA citrate pH 7.8 for 30 min at 95°C and then incubated with mouse to mouse block (ScyTek Laboratories, Logan, Utah, USA), for 10 min at room temperature. Then, sections were incubated with an anti-ATGL rabbit polyclonal primary antibody (Cell Signaling Technology®, cat. number #2439S; 1:100 diluted). Washings were performed with PBS/Tween®20 pH 7.6 (UCS diagnostic, Rome, Italy); reactions were revealed by HRP-DAB Detection Kit (UCS diagnostic). To assess the background of immuno-staining, we included a negative control for each reaction by incubating sections with secondary antibodies (HRP) and a detection system (DAB).

*Human samples.*

Human HCC samples were kindly provided during the whole duration of this study by Prof. Grazi from the Hepato-pancreato-biliary Surgery Unit, Department of Clinical and Experimental Oncology, Regina Elena National Cancer Institute, Rome, Italy. Analyses were performed after approval from the Regina Elena Cancer Institute ethical committee and from the patients. All the samples provided were used in this study. The mean age of the population studied was 69.4 years (range 52-84 years). The mean tumor size was 2.49 cm (range 0.37-5.6 cm) and the mean of total tumor nodules was 2.07 (range 1-6). According to the Edmonson grade, the majority of tumors was partially differentiated (G2, 53.3%) or moderately differentiated (G3, 33.3%); a minority was highly differentiated (G4, 7%) or poorly differentiated (G1, 6.4%). At surgery, livers displayed cirrhosis in the 60% of cases and hepatitis in the 6.7% of cases. HCC was classified as from: alcoholic hepatopathy in the 8% of cases, cirrhosis in the 68% of cases and viral origin in the 24% of cases (HBV 28.6%, HCV 71.4%). Samples were cryopreserved at -80°C and processed as described.

*Bioinformatic analyses.*

*PNPLA2* expression was assessed by Gene Expression Omnibus (GEO; [http://www.ncbi.nlm.nih.gov/geo,](http://www.ncbi.nlm.nih.gov/geo) accession number GSE14520) through an Affymetrix Human Genome Array (>200 HCC *vs* normal livers, NL).

*Cell cultures and treatments.*

Human hepatocellular carcinoma HepG2 (DSMZ no. ACC 180) and Hep3B (DSMZ no. ACC 93) cell lines were obtained from Leibniz-Institut DSMZ, Braunschweig (Germany). Human hepatocellular carcinoma Huh7.5 cell line was a kind gift from Dr. Carla Montesano, Dept. of Biology, University of Rome “Tor Vergata” (Italy). All cell lines were grown in Dulbecco’s modified Eagle’s medium (DMEM) 1g/L glucose (Lonza) supplemented with 10% fetal bovine serum (EuroClone), 2mM L - glutamine (Lonza), 10U/ml penicillin/streptomycin (Lonza) and 1% MycoZap™ (Lonza). Cell lines were authenticated and characterized by the suppliers. Every 6 months, *Mycoplasma test* was carried out according to protocols from our laboratory. Cells were immediately expanded and multiple aliquots were cryopreserved and used within 6 months of resuscitation. Cells were cultured at 37°C in an atmosphere of 5% CO2 in air. During the experiments, cells were plated at a density of 2×105 cells/ml. 2-deoxyglucose (2-DG, Sigma-Aldrich) was used at final concentration of

5

30mM for 24h; 3-bromopyruvate (3BrP, Sigma-Aldrich) was used at a final concentration of 30µM for 24h; doxorubicin (DOXO, Sigma-Aldrich) was used at a final concentration of 2µM for 16h; etoposide (ETO, Sigma-Aldrich) was used at a final concentration of 50µM for 16h; MG-132 (Sigma-Aldrich) was used at a final concentration of 10µM for 4h; GW-7647 (Cayman Chemical) and GW-6471 (Cayman Chemical) were used at a final concentration of 1µM and 5µM, respectively, for 24h; Nutlin-3 (Sigma-Aldrich) was used at a final concentration of 1µM for 24h; C646 (Sigma-Aldrich) was used at a final concentration of 10µM for 24h. Drugs concentration and experimental times were determined after dose-response experiments or on the basis of previous works from our laboratory.

*Plasmids, siRNAs, and transfections.*

The pcDNA™4/HisMaxC (Ev) and the pcDNA™4/HisMaxC-ATGL (ATGL) plasmids were kindly provided by Prof. Rudolf Zechner, Institute of Molecular Biosciences, Karl-Franzens-Universität Graz, Graz (Austria). pEGFP, p*ATGLWT*-EGFP and p*ATGL(Ser47Ala)*-EGFP plasmids were a kind gift of Prof. Daniela Tavian, Laboratory of Human Molecular Biology and Genetics, Università Cattolica del Sacro Cuore, Milan (Italy). pcDNA3.1 and pcDNA3.1-p53WT plasmids were already available in our laboratory. Transient plasmid transfection was carried out 24h after cells plating using Polyethylenimine (PEI) transfection reagent, according to the manufacturer’s instructions. Culture medium was refreshed 24h after transfection. Knockdown of p53 and ATGL was performed by transfecting cells with 10nM SignalSilence® p53 siRNA II (Cell Signaling Technology®) and 10nM Silencer® Select ATGL siRNA (Ambion). Negative control cells were transfected with 10nM SignalSilence® Control siRNA (Unconjugated) (Cell Signaling Technology®) or 10nM Silencer® Select Negative Control #1 siRNA (Ambion), which do not present homology with any other human mRNAs. siRNAs were reversely transfected using Lipofectamine® RNAiMAX Transfection Reagent (ThermoFisher Scientific), according to the manufacturer’s instructions. 24h after siRNA transfection, cells were transfected for ATGL over-expression as previously described. 24h hours after plasmid transfection, cells were treated as indicated in the experiments.

*Western blot analyses.*

Total protein lysates were obtained from cells by 30-min on ice rupturing incubation in Lysis Buffer (50mM Tris-HCl, pH 7.4, 150mM NaCl, 1mM EDTA, 1% Triton X-100, 0.5% Sodium Deoxycholate, 0.1% SDS, 10mM NaF, 5mM Sodium Pyrophosphate, 2mM Sodium Orthovanadate, protease inhibitor cocktail, Roche Applied Science) followed by sonication and centrifugation at 10,000×*g* for 20 min at 4°C. Protein concentration was determined according to the Lowry’s method and protein extracts were electrophoresed by SDS-PAGE and blotted onto nitrocellulose membrane (Bio-Rad). Primary antibodies used are as follows: ACO2 (abcam, cat. number ab110321), ATGL (Cell Signaling Technology®, cat. number #2138S), CASP-9 (Santa Cruz Biotechnology, cat. number sc-56076), COX IV (abcam, cat. number ab14744), FH (Cell Signaling Technology®, cat. number #4567S), H3 (abcam, cat. number ab1791), Ac-H3K56 (Merck, cat. number 07-677-I), HK-2 (abcam, cat. number ab104836), HSP60 (Santa Cruz Biotechnology, cat. number sc-13115), IDH2 (Cell Signaling Technology®, cat. number #56439S), Lamin B1 (Santa Cruz Biotechnology, cat. number sc-377000), LDH (Santa Cruz Biotechnology, cat. number sc-33781), MDM2 (Santa Cruz Biotechnology, cat. number sc-965), NDUFB8 (abcam, cat. number ab110242), p300 (Santa Cruz Biotechnology, cat. number sc-585), p53 (Sigma Aldrich, cat. number P5813),

p53-AcK382 (Cell Signaling Technology®, cat. number #2525S), p53-pSer15 (Cell Signaling Technology®, cat. number #9284S), PARP-1 (Cell Signaling Technology®, cat. number #9532S), PGC-1α (Santa Cruz Biotechnology, cat. number sc-517380), PPAR-α (Santa Cruz Biotechnology, cat. number sc-398394), SDH-A (abcam, cat. number ab14715), SIRT1 (Cell Signaling Technology®, cat. number #9475S), TOMM20 (Santa Cruz Biotechnology, cat. number sc-11021), Ubiquitinated proteins (abcam, cat. number ab7780), β-Actin (Cell Signaling Technology®, cat. number #4970S), γ-H2AX (Cell Signaling Technology®, cat. number #9718S). The specific protein complex, formed upon incubation with specific secondary antibodies (Bio-Rad), was identified using a Fluorchem Imaging System (Alpha Innotech) after incubation with LiteAblot® TURBO (EuroClone). Fluorchem Imaging System (Alpha Innotech) was also used to perform densitometry analyses.

*Nuclear fraction isolation.*

Nuclear extracts were obtained as previously described 4. For purity determination, total cell extracts and cytosolic and nuclear fractions were analyzed by Western blot with antibodies raised against LDH (specific for post-nuclear fraction; Santa Cruz Biotechnology, cat. number sc-33781) and Lamin B1 (specific for nuclei; Santa Cruz Biotechnology, cat. number sc-377000).

*Quantitative real-time PCR (RT-qPCR).*

Cells were homogenized in TRI Reagent, and RNA was extracted according to manufacturer’s instructions. For RNA extraction from human and mice samples, 25mg of tissue were minced in TRI Reagent (1:40, w/v) on ice by 30 strokes using a Potter-Elvehjem automatic homogenizer. To generate cDNA for RT-qPCR, total RNA was solubilized in ribonuclease-free water and 1µg of total RNA used for reverse transcription by PrimeScript™ RT Reagent Kit (Perfect Real Time) (Takara). To selectively hybridize unique regions of the appropriate gene sequence, specific primer pairs were designed and tested with primer-BLAST (NCBI). Primers used were obtained from Sigma-Aldrich and are as follows:

| Gene | Species | Forward Primer | Reverse Primer |
| --- | --- | --- | --- |
| *Actb* | Mouse | 5’-CACACCCGCCACCAGTTCGC-3’ | 5’-TTGCACATGCCGGAGCCGTT-3’ |
| *Afp* | Mouse | 5’-AAACCTCCAGGCAACAACCA-3’ | 5’-TTCCTTGGCAACACTCCTCG-3’ |
| *Pnpla2* | Mouse | 5’-TCGTGTTTCAGACGGAGAGAA-3’ | 5’-CAGACATTGGCCTGGATGAG-3’ |
| *ACAA2* | Human | 5’-CAGGGAATGCATCGGGTGTA-3’ | 5’-GCCCACAATTCTTGCCAGTG-3’ |
| *ACADM* | Human | 5’-CGGGGTTCGGGCGATG-3’ | 5’-CTGCTGTTCGGTGAACTCAAA-3’ |
| *ACADS* | Human | 5’-CCTCAGCGAACCAGGGAAC-3’ | 5’-TTCAGAACCCATGAGTCGCC-3’ |
| *ACC1* | Human | 5’-GGTGAAGAGGGTGCGTTTCA-3’ | 5’-CCAAAAAGACCTAGCCCTCAAG-3’ |
| *ACO2* | Human | 5’-ACAGCCTACTGGTGACTCGG-3’ | 5’-GGGCTCAAAGTGGCTCATC-3’ |
| *ACTB* | Human | 5’-GGCCGAGGACTTTGATTGCA-3’ | 5’-GGGACTTCCTGTAACAACGCA-3’ |
| *ATP5J* | Human | 5’-GGGCATGCAGACGGTTAATG-3’ | 5’-AAGCCCATCCATGCGGTTTA-3’ |
| *ATP5O* | Human | 5’-CGCGTTTCTCTCTTCCCACT-3’ | 5’-TACCTGAACAGGAGGCCTCA-3’ |
| *ATP6* | Human | 5’-CACACCTACACCCCTTATCCC-3’ | 5’-TCATTATGTGTTCTCGTGCAG-3’ |
| *COX4I1* | Human | 5’-CCCGGCATTTTACGACGTTC-3’ | 5’-CAACATTCTGCCGCCACTG-3’ |
| *COX5A1* | Human | 5’-GGGCATGCAGACGGTTAAATG-3’ | 5’-CAACATTCTGCCGCCACTG-3’ |

| *Cpt1A* | Human | 5’-ACAGTCGGTGAGGCCTCTTA-3’ | 5’-CCACCAGTCGCTCACGTAAT-3’ |
| --- | --- | --- | --- |
| *Cpt2* | Human | 5’-ATTTCGGGACCCTGGTTTGA-3’ | 5’-CCACCAGTCGCTCACGTAAT-3’ |
| *CS* | Human | 5’-TCCGACCCTTACCTGTCCTT-3’ | 5’-ACTTCCTGATTTGCCAGTCC-3’ |
| *CYB* | Human | 5’-TGAAACTTCGGCTCACTCCT-3’ | 5’-AATGTATGGGATGGCGGATA-3’ |
| *CYC* | Human | 5’-TACGGACACCTCAGGCAGT-3’ | 5’-CACGGTGAGACCACGGATAG-3’ |
| *ECHS1* | Human | 5’-GCCTCGGGTGCTAACTTTGA-3’ | 5’-GCCATCGCAAAGTGCATTGA-3’ |
| *EP300* | Human | 5’-TGCAGGCATGGTTCCAGTTT-3’ | 5’-AGGTAGAGGGCCATTAGAAGTCA-3’ |
| *FASN* | Human | 5’-GACCGCTTCCGAGATTCCAT-3’ | 5’-GAGGCCTATCTGGATGGCAG-3’ |
| *FH* | Human | 5’-TGAATGTTTTCAAGCCAATGAT-3’ | 5’-CCACCACGCAGTTTTCTGTA-3’ |
| *IDH2* | Human | 5’-CCATCATCTGCAAAAACATCC-3’ | 5’-CCAATGGTGATGGGCTTG-3’ |
| *MDH2* | Human | 5’-CAGGACCAGCTGACAGCAC-3’ | 5’-AGCCTGCTCCGGCTTTAG-3’ |
| *MDM2* | Human | 5’-GATGGTGAGGAGCAGGCAAA-3’ | 5’-AGGGTCTCTTGTTCCGAAGC-3’ |
| *MtCO1* | Human | 5’-GATTTTTCGGTCACCCTGAAG-3’ | 5’-CTCAGACCATACCTATGTATC-3’ |
| *ND1* | Human | 5’-CTACTACAACCCTTCGCTGAC-3’ | 5’-GGATTGAGTAAACGGCTAGGC-3’ |
| *NDUFB6* | Human | 5’-TGGTCCATGGGGTATACAAAA-3’ | 5’-TCTCCAGAATTGTATCACCAGG-3’ |
| *NDUFV1* | Human | 5’-GCGGGTATCTGTGCGTTTC-3’ | 5’-GAACCTTTCAGCCTCCAGTCA-3’ |
| *PNPLA2* | Human | 5’-ACCAGCATCCAGTTCAACCT-3’ | 5’-ATCCCTGCTTGCACATCTCT-3’ |
| *SCO2* | Human | 5’-CTTCCTCTCGTGCTTGGTCC-3’ | 5’-ATGGATCTGATGCTCCTCGC-3’ |
| *SDH-A* | Human | 5’-TGGGAACAAGAGGGCATCTG-3’ | 5’-CCACCACTGCATCAAATTCAT-3’ |
| *SIRT1* | Human | 5’-AGGCCACGGATAGGTCCAT-3’ | 5’-CTCAGGTGGAGGTATTGTTTCC-3’ |
| *SLC16A1* | Human | 5’-GGCTGGGCAGTGGTAATTGGAGCT-3’ | 5’-GGCCCGATTGGTCGCATGAGGGCT-3’ |
| *SLC2A1* | Human | 5’-TTCACTGTCGTGTCGCTGTT-3’ | 5’-TGAGTATGGCACAACCCGC-3’ |
| *SLC2A2* | Human | 5’-GACCACGTCCTGCTGCTTTA-3’ | 5’-GGTCCACAGAAGTCCGCAAT-3’ |
| *SUCGL2* | Human | 5’-AGCCAGCCAACTTCTTGGA-3’ | 5’-GGATGGCTTCAACCTTAGGA-3’ |
| *TP53* | Human | 5’-ATCTACTGGGACGGAACAGC-3’ | 5’-GTGAGGCTCCCCTTTCTTG-3’ |
| *UQCRC1* | Human | 5’-GGATATGGCCCCATTGAGCA-3’ | 5’-CCGAAGTGCTGTGTTTGTGG-3’ |

Real-time qPCR was performed using the SYBR® *Premix Ex Taq* (Tli RNase H Plus) (Takara) on a StepOne real-time PCR System (Applied Biosystems). All reactions were run as triplicates. Data were analyzed by the StepOne™ Software (v2.3) using the second-derivative maximum method. The fold changes in mRNA levels were relative to a control after normalization to the internal standard *ACTB* (for cells and human samples) and *Actb* (for mouse specimens).

*Chromatin Immunoprecipitation (ChIP).*

Chromatin immunoprecipitation assays were performed on nuclear lysates as previously described

5. Briefly, cells were cross-linked for 10 min at room temperature with 1% final concentration of 37% formaldehyde and reaction was stopped by 5 min incubation in 125mM glycine. Cell monolayer was harvested by scraping in ice-cold PBS containing protease inhibitors and nuclei isolated as described before. Chromatin was sonicated using Bioruptor NextGen (Diagenode) to High Power, 18 cycles for 30 seconds ON, 30 seconds OFF. Average size of sonicated DNA was

around 500-1000 bp, as measured by agarose gel electrophoresis. Samples were pre-cleared with pre-adsorbed Salmon Sperm G-coupled Sepharose beads, and overnight immunoprecipitated with anti-PPAR-α (Santa Cruz Biotechnology, cat. number sc-398394) or anti-p53 (Sigma Aldrich, cat. number P5813) antibodies. DNA amplification was performed using SYBR® Premix Ex Taq (Tli RNase H Plus) (Takara) on a StepOne real-time PCR System (Applied Biosystems). All reactions were run as triplicates. Data were analyzed by the StepOne™ Software (v2.3) using the second- derivative maximum method. Results are expressed as fold enrichment with respect to IgG control. Primers used are as follows:

| Gene | Forward Primer | Reverse Primer |
| --- | --- | --- |
| *EP300* | 5’-CTACACATTTGGCAGCAGCA-3’ | 5’-GCTTTGACCTAGCTCAGCTTTC-3’ |
| *SLC16A1* | 5’-GAGCCTACGAGGTCGGTTCT-3’ | 5’-GCTGGGATGTGTTTGGATTC-3’ |
| *SLC2A1* | 5’-GGAGACAGGGAAGGGAGAAG-3’ | 5’-CAGCGTCTACTGCATGGAAA-3’ |

*Cell proliferation assays.*

Cell proliferation was evaluated by Trypan blue staining procedure, CCK-8 colorimetric assay using the Cell Counting Kit-8 (CCK-8, Dojindo Molecular Technologies) according to manufacturer’s protocol and by Bromodeoxyuridine (BrdU) incorporation assay. For BrdU incorporation assay, cells were treated with 10µM BrdU for 4h before the end of the experiments. Then, cells were fixed with an ethanol:acetic acid:di-deionized water solution (18:1:1, v/v/v) for 30 min. DNA was denaturized with a first 10 min incubation on ice with 1N HCl and a second 10 min incubation on ice with 2N HCl. Cells were then permeabilized with a PBS/0.4% Triton X-100 solution for 10 min, blocked with PBS/10% goat serum solution for 1h and incubated overnight with an anti-BrdU (Santa Cruz Biotechnology, cat. number sc-32323) antibody and then for 1h with an Alexa Fluor™ 568 donkey anti-mouse IgG (H+L) secondary antibody; nuclei were stained with 1µg/ml of Hoechst 33342 for 10 min. Fluorescent images of cells were digitized with a Delta Vision Restoration Microscopy System (Applied Precision, Issaquah, WA) equipped with an Olympus IX70fluorescence microscope (Olympus Italia, Segrate, Milano, Italy).

*Oil Red-O staining.*

Oil Red-O staining was performed as follows. Culture medium was removed and cells rinsed in PBS. Cells were fixed twice with paraformaldehyde for 10 min and 1h at room temperature without intermediate washes. Paraformaldehyde was removed and rinsed with PBS. Cells were incubated for 5 min at room temperature with 60% isopropanol. After complete drying, cells were incubated for 10 min with the Oil Red-O working solution, obtained from a filtered 6:4 dilution of a pre-filtered 0.35% Oil Red-O stock solution in 100% isopropanol with di-deionized water. Cells were washed with di-deionized water and completely dried. Oil Red-O incorporated in lipid droplets was eluted with a unique wash in 100% isopropanol for 10 min at room temperature by gentle shaking and relative absorbance measured at 510nm with an Eppendorf BioSpectrometer®.

*Measure of triglyceride content.*

Triglycerides content was measured with a Triglyceride Quantification Colorimetric/Fluorometric Kit (BioVision Incorporated), according to the manufacturer’s protocol. Values were normalized on protein.

*Assessment of mitochondrial function.*

Seahorse XF96e Analyzer (Seahorse Bioscience® XP Technology Agilent Santa Clara CA, USA) was used to determine cell’s mitochondrial functionality (Cell Mito Stress Test) and endogenous/exogenous fatty acids oxidation (FAO test). All procedures were performed according to the manufacturer's instructions. Briefly, HepG2 cells were seeded at a density of 7.5x104 per well into on micro-lysine-coated XF96 microplates in 100μl DMEM 10% FBS and transfected as previously described. To perform Cell Mito Stress Test, medium was replaced with XF Base supplemented with 1mM pyruvate, 2mM glutamine and 10mM glucose, and cells were incubated in a CO2 free incubator at 37°C for 45 min. Seahorse FluxPak cartridge was equilibrated and the corresponding injection ports were loaded with the mito-stressor agents Oligomycin (1μg/ml), FCCP (1μM) and Rotenone/Antimycin A (0.5μM). To perform the FAO test, medium was replaced with a Substrate-Limited Medium for 4h, then cells were incubated in a CO2 free incubator at 37°C with XF FAO assay medium supplemented with XF BSA, XF BSA-Etomoxir (40μM), XF Palmitate-BSA and XF Palmitate-BSA-Etomoxir (40μM), respectively (Seahorse Bioscience®). Seahorse FluxPak cartridge was equilibrated and the corresponding injection ports were loaded with the mito- stressor agents Oligomycin (3μg/ml), FCCP (1μM) and Antimycin A/Rotenone (2μM). Oxygen Consumption Rate (OCR) was measured and obtained results were analysed using *XFe Wave Software* (Seahorse Bioscience).

*Oxygen consumption and ATP measurement.*

Oxygen consumption was determined at 37°C using a Clark-type oxygen electrode equipped with thermostatic control and magnetic stirring. Cells were washed in PBS, collected and centrifuged at 800×g for 10 min. Cell pellets were suspended in fresh culture medium previously equilibrated at 37°C in an atmosphere of 5% CO2. For each sample, oxygen consumption was measured for 15 min and ΔO2 values normalized on cells. For ATP measurement, cells were lysed and incubated in 100mM Tris-HCl, pH 7.75, 4mM EDTA for 2 min at 100°C. ATP levels were measured by the ATP Bioluminescence Assay Kit CLS II (Roche Applied Science) using a microplate luminometer (Perkin Elmer) after incubation with the luciferin/luciferase reagents. Data were normalized on protein.

*Determination of mitochondrial mass.*

One hour before the end of the experimental time, cells were incubated with 250nM MTG, washed with PBS, collected, resuspended in ice-cold PBS, and the fluorescence intensity immediately analyzed cytofluorometrically by recording FL-1 fluorescence by means of a FACScalibur instrument. 10,000 events were counted and data expressed as arbitrary units.

*Extracellular lactate assay.*

Extracellular lactate assay was performed as previously described 1. Briefly, 500µl of cell medium were precipitated with 250µl of 30% trichloroacetic acid. Media were frozen and then centrifuged at 14,000×g for 20 min at 4°C. 10µl of supernatant were incubated for 30 min at 37°C in 290µl of reaction buffer (0.2M glycine/hydrazine buffer, pH 9.2, 0.6 mg/ml NAD+ and 17 U/ml LDH).

10

NAD+ reduction was followed at 340 nm using an Eppendorf BioSpectrometer® and nmoles of NADH formed were considered stoichiometrically equivalent to extracellular lactate. Thus, relative absorbance values were converted to lactate concentration using an extinction coefficient of 6,220 M-1cm-1 at 340nm for NADH. Concentrations were normalized on total proteins.

*Measurement of 2-NBDG uptake.*

One hour before the end of the experimental time, cells were incubated with 100µM 2-NBDG, a fluorescent derivative of 2-deoxy-D-glucose, washed with PBS, collected, resuspended in ice-cold PBS, and the fluorescence intensity immediately analyzed cytofluorometrically by recording FL-1 fluorescence by means of a FACScalibur instrument. 10,000 events were counted and data expressed as arbitrary units.

*Measurement of HK activity.*

HK activity was determined spectrophotometrically. Cells were wash in PBS and lysed for 30 min on ice in lysis buffer (50mM Tris-HCl, pH 7.5, 1mM EDTA, 150mM NaCl, 1% NP-40, 1mM DTT, protease inhibitor cocktail). 50µg of total protein lysate was incubated in reaction buffer (50mM Tris-HCl, pH 7.5, 10mM MgCl2, 0.6mM ATP, 100mM glucose, 0.2mM NADP+, 0.1U/mL of glucose-6- phosphate dehydrogenase) for 30 min at 37°C and optical absorbance measured at 340nm every 15 sec for 10 min with an Eppendorf BioSpectrometer®. Enzyme activity was represented as change in absorbance per minute (U), normalized on protein.

*Measurement of SIRT1 activity.*

SIRT1 activity was determined by means of the SIRT1/Sir2 Deacetylase Fluorometric (Human) Assay Kit (Abnova), according to the manufacturer’s protocol.

*Fluorescence microscopy analyses.*

Cell medium was removed and cells rinsed with PBS and fixed with an ethanol:acetic acid:ddH2O (18:1:1, v/v/v) for 30 min, permeabilized with a PBS/0.4% Triton X-100 solution for 10min, blocked with PBS/10% goat serum solution for 1h and incubated overnight with an anti-p53 (Sigma Aldrich, cat. number P5813) antibody and then for 1h with an Alexa Fluor™ 568 donkey anti-mouse IgG (H+L) secondary antibody; nuclei were stained with 1µg/ml of Hoechst 33342 for 10 min. Fluorescent images of cells were digitized with a Delta Vision Restoration Microscopy System (Applied Precision, Issaquah, WA) equipped with an Olympus IX70fluorescence microscope (Olympus Italia, Segrate, Milano, Italy).

*Cell viability.*

The percentages of dead and viable cells were evaluated by Trypan blue staining and by Propidium Iodide (PI) procedures. For PI procedure, cells were trypsinized, centrifuged at 1,200×g for 10 min at 4°C and resuspended in PI Buffer (50µg/ml Propidium Iodide, 0.1% sodium citrate, 0.1% Triton X-100, 200µg/ml RNAse) and stained for 15 min at room temperature. Fluorescence intensity was cytofluorometrically analyzed by recording FL-2 fluorescence by means of a FACScalibur instrument. Dead cells were expressed as percentage of subG1 cells.

*Data analysis.*

Data were from at least 3 independent experiments, unless otherwise indicated. Results are presented as means ± SD. Statistical evaluation was appropriately conducted by unpaired 2-tailed Student *t* test, Fisher’s exact test, one- or two-way ANOVA, using the GraphPad Prism 7 software, according to the experiment. Bonferroni post-test was used to identify individual differences when ANOVA tests were significant. Comparisons were statistically considered significant at *p*≤0.05 (*), very statistically significant at *p*≤0.01 (**) and extremely statistically significant at *p*≤0.001 (***). Sample size and statistics are reported in every figure and were determined according to published literature, together with the sufficient power. No experiment was randomized and performed blindly.

# Supplementary References.

1. Desideri E, Vegliante R, Cardaci S, Nepravishta R, Paci M, Ciriolo MR. MAPK14/p38alpha-dependent modulation of glucose metabolism affects ROS levels and autophagy during starvation. Autophagy 2014; 10: 1652-1665.
2. Raubenheimer PJ, Nyirenda MJ, Walker BR. A choline-deficient diet exacerbates fatty liver but attenuates insulin resistance and glucose intolerance in mice fed a high-fat diet. Diabetes 2006; 55: 2015-2020.
3. Tolba R, Kraus T, Liedtke C, Schwarz M, Weiskirchen R. Diethylnitrosamine (DEN)-induced carcinogenic liver injury in mice. Lab Anim 2015; 49: 59-69.
4. Vegliante R, Desideri E, Di Leo L, Ciriolo MR. Dehydroepiandrosterone triggers autophagic cell death in human hepatoma cell line HepG2 via JNK-mediated p62/SQSTM1 expression. Carcinogenesis 2016; 37: 233-244.
5. Verdone L, La Fortezza M, Ciccarone F, Caiafa P, Zampieri M, Caserta M. Poly(ADP-Ribosyl)ation Affects Histone Acetylation and Transcription. PloS one 2015; 10: e0144287.
