# Supplementary figures and images for: Forcing ATGL expression in hepatocarcinoma cells imposes glycolytic rewiring through PPAR-α/p300-mediated acetylation of p53
Source: Oncogene. 2018 Oct 26;38(11):1860–75. doi: 10.1038/s41388-018-0545-0 (PMC6756110; doi:10.1038/s41388-018-0545-0)

Figure S1

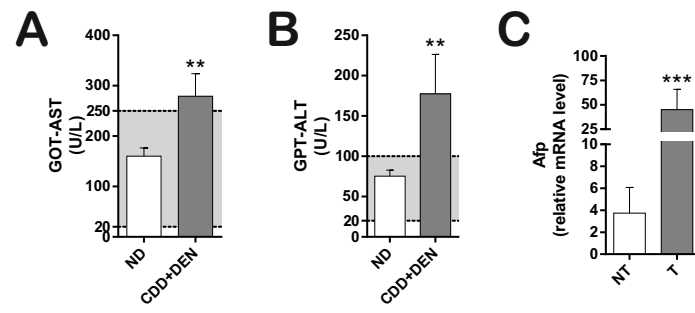

Supplement: Supplementary file 2 — Supplementary Figure 1 [file 41388_2018_545_MOESM2_ESM.pdf]

# Figure S2

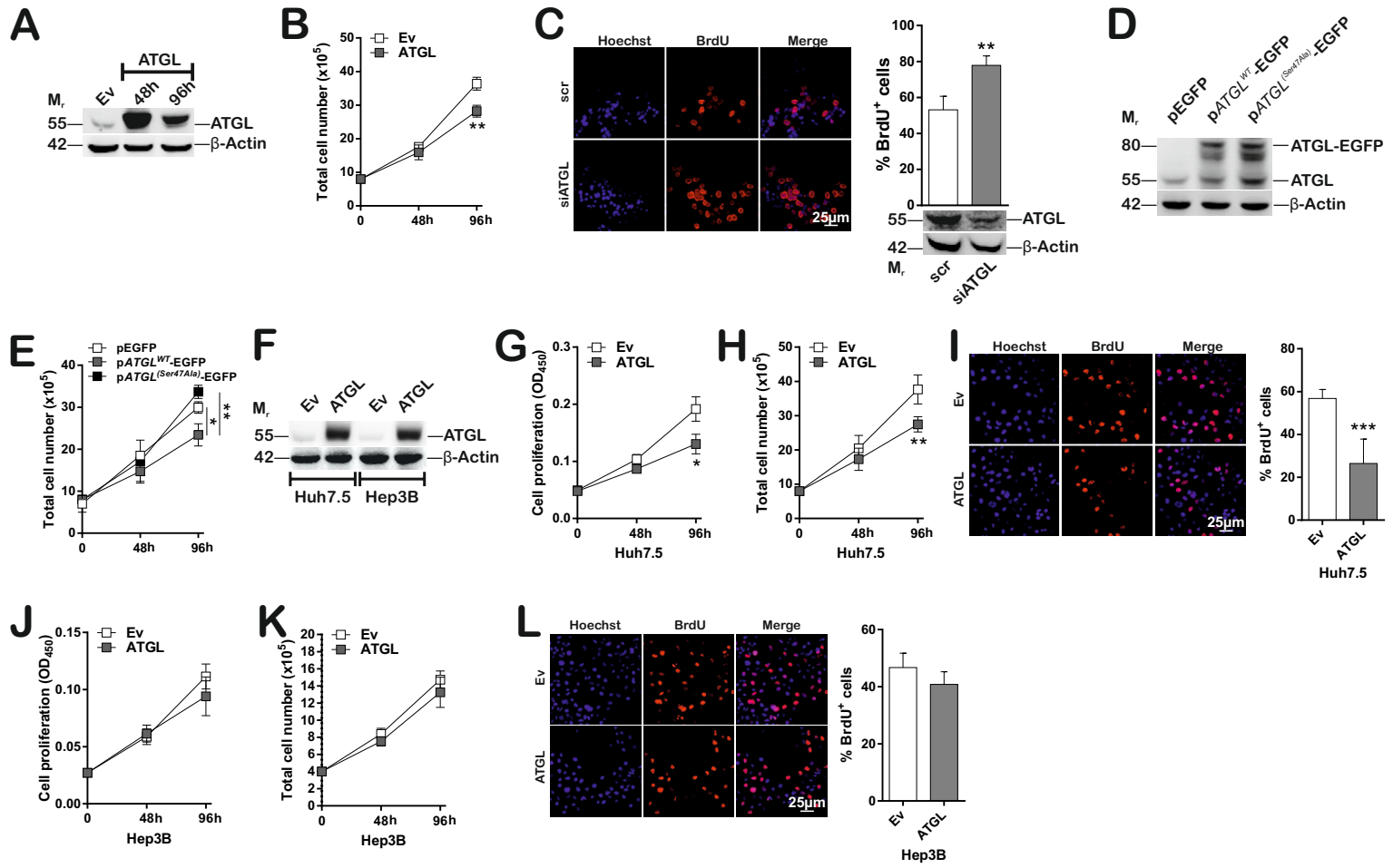

Supplement: Supplementary file 3 — Supplementary Figure 2 [file 41388_2018_545_MOESM3_ESM.pdf]

# Figure S3

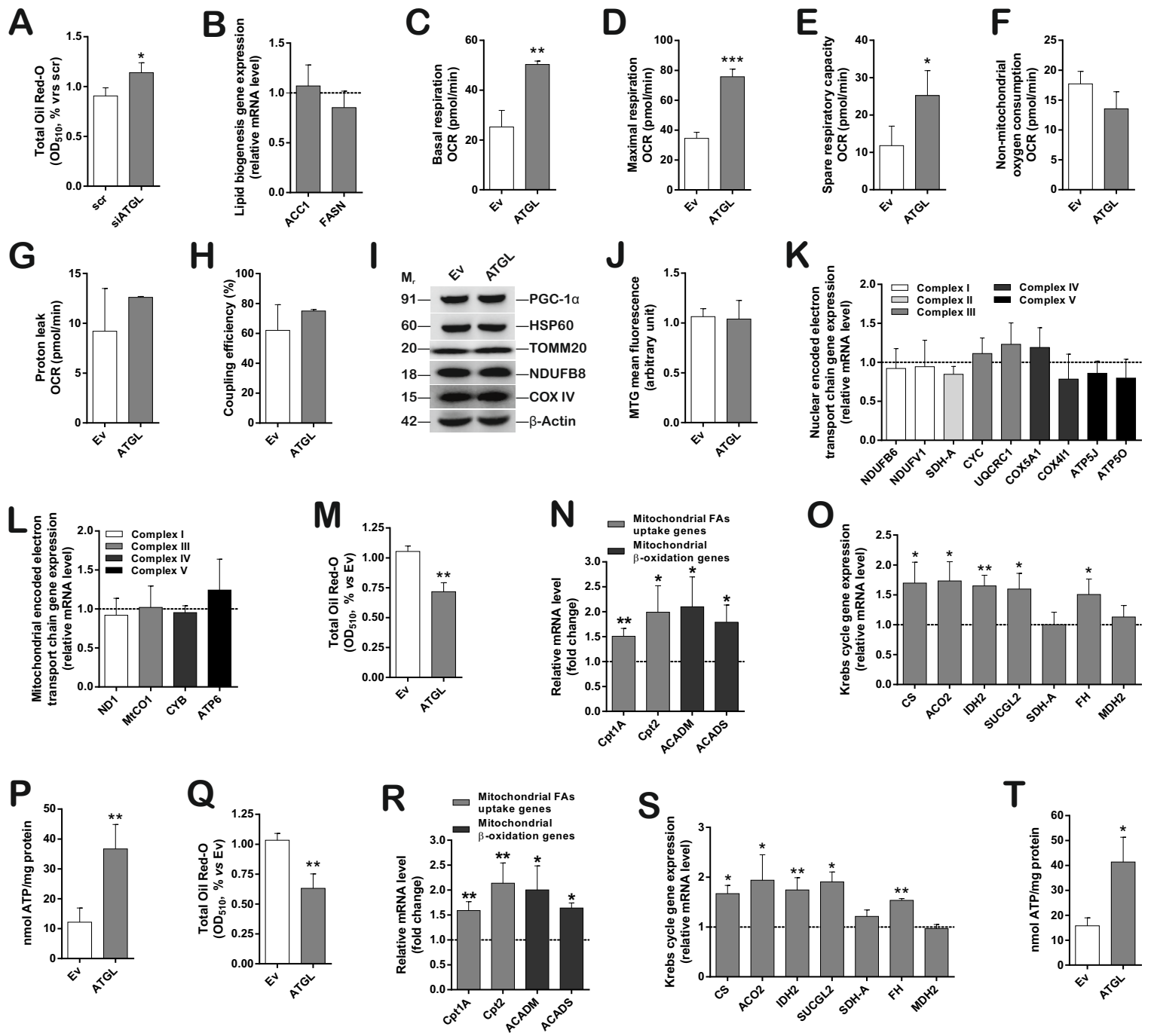

Supplement: Supplementary file 4 — Supplementary Figure 3 [file 41388_2018_545_MOESM4_ESM.pdf]

Figure S4

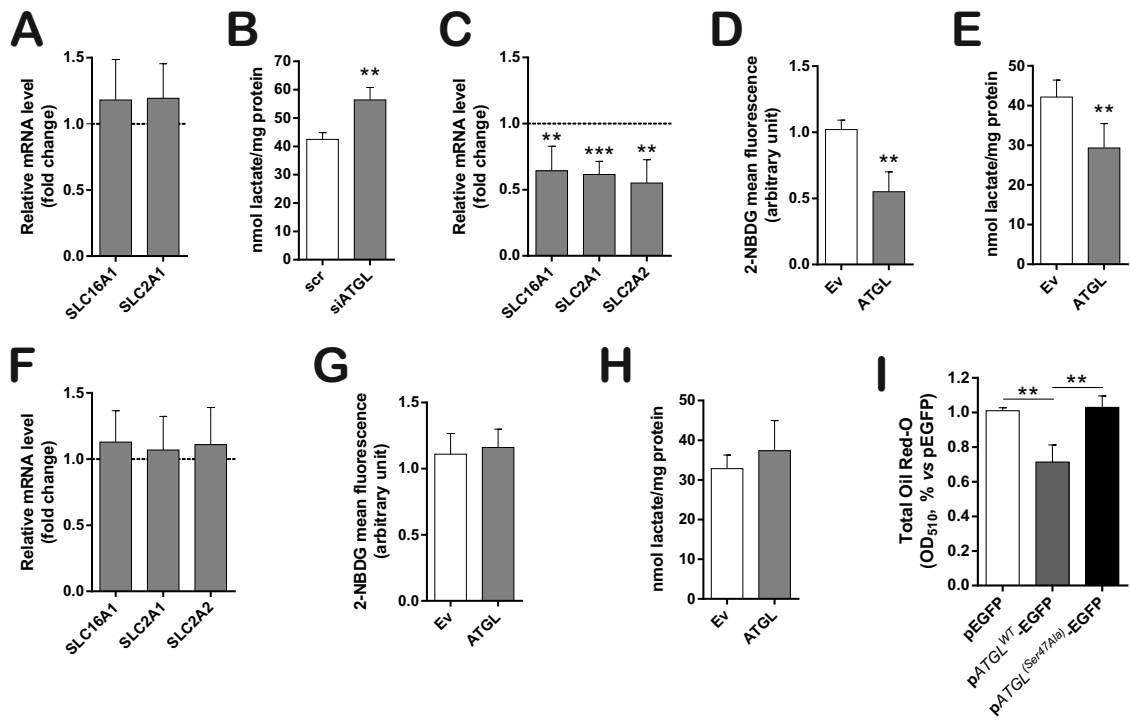

Supplement: Supplementary file 5 — Supplementary Figure 4 [file 41388_2018_545_MOESM5_ESM.pdf]

# Figure S5

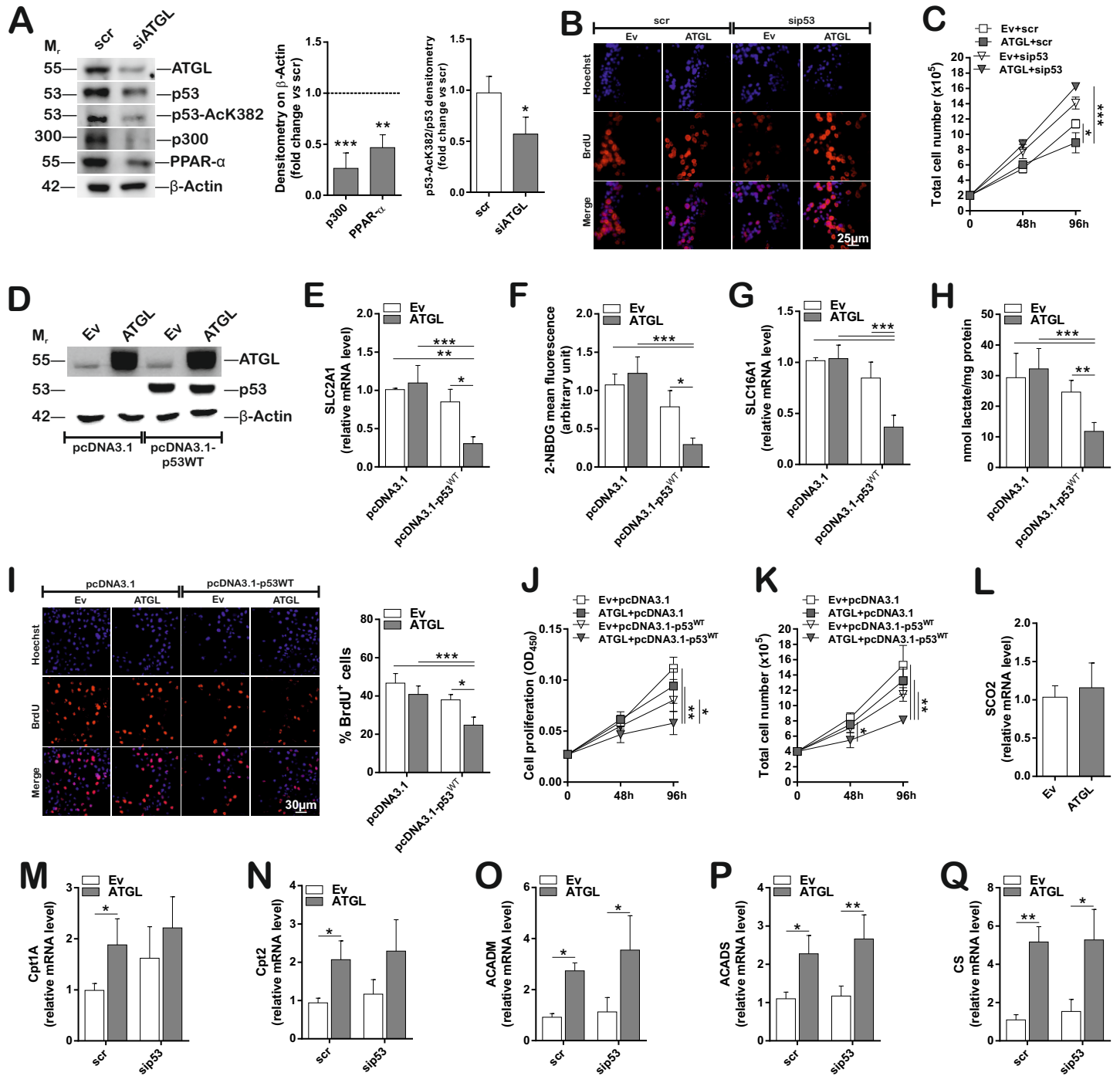

Supplement: Supplementary file 6 — Supplementary Figure 5 [file 41388_2018_545_MOESM6_ESM.pdf]

# Figure S6

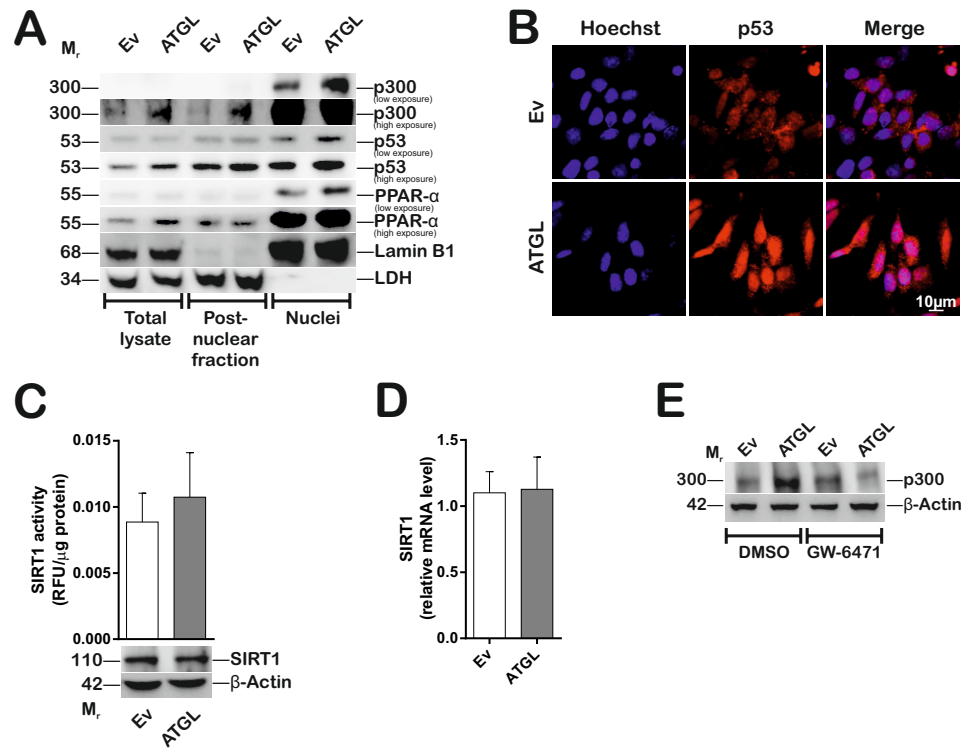

Supplement: Supplementary file 7 — Supplementary Figure 6 [file 41388_2018_545_MOESM7_ESM.pdf]

# Figure S7

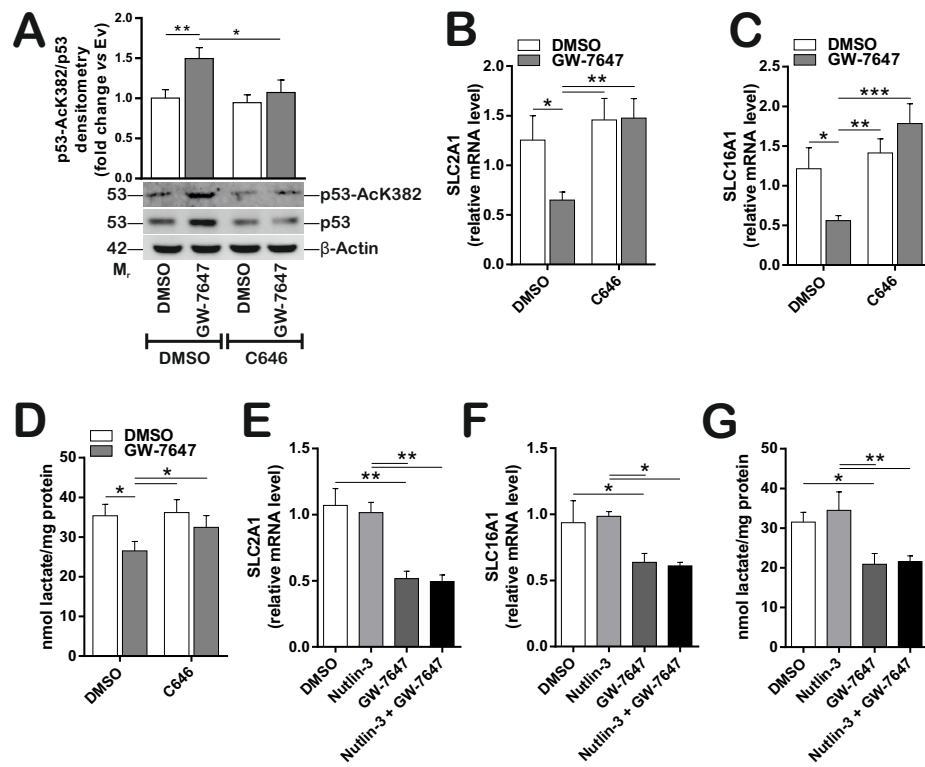

Supplement: Supplementary file 8 — Supplementary Figure 7 [file 41388_2018_545_MOESM8_ESM.pdf]
